# Learning the payoffs and costs of actions

**DOI:** 10.1371/journal.pcbi.1006285

**Published:** 2019-02-28

**Authors:** Moritz Möller, Rafal Bogacz

**Affiliations:** MRC Brain Network Dynamics Unit, Nuffield Department of Clinical Neurosciences, University of Oxford, Oxford, United Kingdom; École Normale Supérieure, College de France, CNRS, FRANCE

## Abstract

A set of sub-cortical nuclei called basal ganglia is critical for learning the values of actions. The basal ganglia include two pathways, which have been associated with approach and avoid behavior respectively and are differentially modulated by dopamine projections from the midbrain. Inspired by the influential opponent actor learning model, we demonstrate that, under certain circumstances, these pathways may represent learned estimates of the positive and negative consequences (payoffs and costs) of individual actions. In the model, the level of dopamine activity encodes the motivational state and controls to what extent payoffs and costs enter the overall evaluation of actions. We show that a set of previously proposed plasticity rules is suitable to extract payoffs and costs from a prediction error signal if they occur at different moments in time. For those plasticity rules, successful learning requires differential effects of positive and negative outcome prediction errors on the two pathways and a weak decay of synaptic weights over trials. We also confirm through simulations that the model reproduces drug-induced changes of willingness to work, as observed in classical experiments with the D2-antagonist haloperidol.

## Author summary

The basal ganglia are structures underneath the surface of the vertebrate brain, associated with error-driven learning. Much is known about the anatomical and biological features of the basal ganglia; scientists now try to understand the algorithms implemented by these structures. Numerous models aspire to capture the learning functionality, but many of them only cover some specific aspect of the algorithm. Instead of further adding to that pool of partial models, we unify two existing ones—one which captures what the basal ganglia learn, and one that describes the learning mechanism itself. The first model suggests that the basal ganglia weigh positive against negative consequences of actions according to the motivational state. It hints how payoff and cost might be represented, but does not explain how those representations arise. The other model consists of biologically plausible plasticity rules, which describe how learning takes place, but not how the brain makes use of what is learned. We show that the two theories are compatible. Together, they form a model of learning and decision making that integrates the motivational state as well as the learned payoffs and costs of opportunities.

## Introduction

What guides rational behavior in a complex environment? Certainly, knowledge of the typical payoffs and costs of acting a certain way is critical for successful action selection. Those payoffs and costs do not only depend on the action that is carried out, but also on the environmental state, henceforth referred to a ‘situation’. If payoffs and costs are represented separately in the animal’s brain, they can be weighted depending on animal’s motivational (i.e. internal) state, which can vary independently of the environmental situation. For example, consider the action ‘harvesting fruit from a tree’ in the situation ‘close to a fruit-bearing tree’. It has a payoff connected with the nutrients in the fruit, but also costs related to the effort, the risk of pain and the exposure to predators associated with climbing a tree. The nutrients in the fruit are only valuable for the animal if it is hungry, i.e. if it is in the appropriate internal state. So, when it is hungry, the payoffs of climbing a tree which was identified as fruit-bearing should be weighted more than the costs, to ensure that the animal searches for food. By contrast, when the animal is not hungry at all, the payoffs should be weighed less than the costs, to make sure that it does not climb the tree without necessity. In summary, the payoffs and costs of a specific action (‘climbing a nearby tree’) carried out in a certain environmental situation (‘near fruit-bearing tree’) should be weighed against each other according to the motivational state (‘hunger’) to correctly asses the action’s utility.

**Fig 1:**
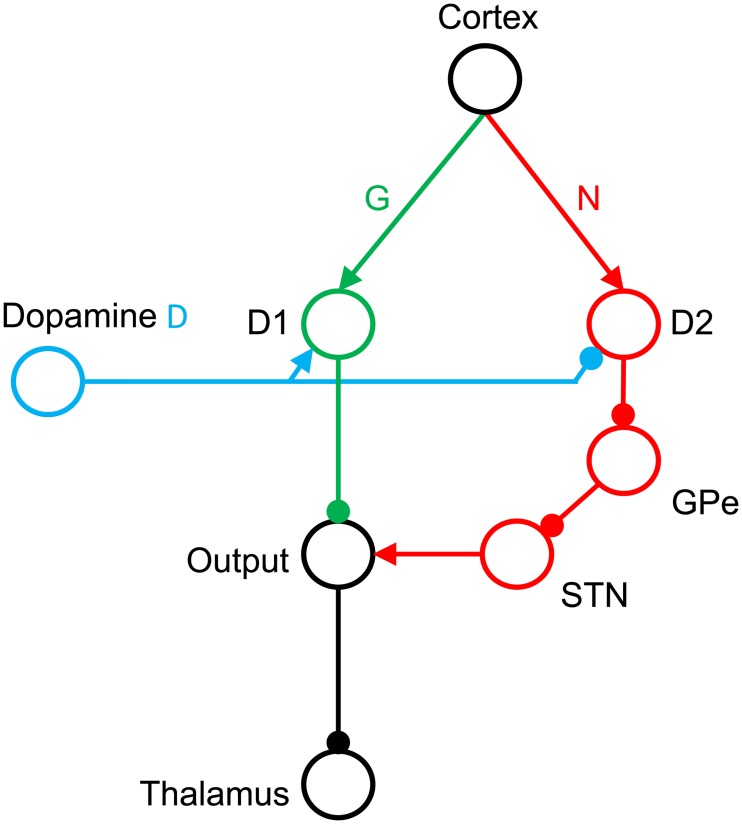
The organization of the basal ganglia. Circles denote neural populations in the areas indicated by labels next to them, where D1 and D2 correspond to striatal neurons expressing D1 and D2 receptors respectively, STN stands for the subthalamic nucleus, GPe for the external segment of globus pallidus, and Output for the output nuclei of the basal ganglia, i.e. the internal segment of globus pallidus and the substantia nigra pars reticulata. Arrows and lines ending with circles denote excitatory and inhibitory connections respectively.

In all vertebrates, an important role in this process of action evaluation and selection is played by a set of subcortical structures called the basal ganglia [1]. The basal ganglia are organized into two main pathways shown schematically in green and red in Fig 1. The Go or direct pathway is related to the initiation of movements, while activation of the No-Go or indirect pathway results in targeted movement inhibition [2]. These two pathways include two separate populations of striatal neurons expressing different dopaminergic receptors [3]. The striatal Go neurons express D1 receptors and are excited by dopamine, while the striatal No-Go neurons express D2 receptors and are inhibited by dopamine [4]. Thus dopamine changes the balance between the two pathways and promotes action initiation over inhibition.

The competition between Go and No-Go pathways during action selection and the role of dopaminergic modulation are subject of many interpretations and models, e.g. [5–7]. In particular, the Opponent Actor Learning (OpAL) hypothesis suggests that the Go and No-Go neurons specialise in encoding the values of actions with positive or negative consequences respectively [8]. We extend the OpAL hypothesis further by proposing that for each individual action, the direct and indirect pathway separately encode the learned positive and negative consequences. As the dopaminergic neurons modulate the Go and No-Go neurons in opposite ways, dopamine controls the extent to which positive and negative consequences affect the activity in the thalamus, through the output of the basal ganglia [8]. For example, when motivation is high, the dopaminergic neurons will excite the Go neurons and inhibit the No-Go neurons. Consequently, positive action values will influence the decision more than negative action values. By contrast, when the motivation is low, the Go neurons tend to be excited to a smaller degree, but the No-Go neurons will be released from inhibition, such that negative values are weighted stronger.

Much research has also focused on how the synapses of Go and No-Go neurons are modified by experience. A systematic investigation revealed that bursts of activity of dopaminergic neurons encode outcome prediction errors, which measure the difference between outcome (typically rewards) obtained and expected [9, 10]. Note that we use the phrases ‘outcome prediction error’ and ‘reinforcement’ instead of the more common ‘reward prediction error’ and ‘reward’ respectively. This use of language emphasizes that in our theory, the feedback signal is informative of both positive and negative events and that not only rewards but any outcome will be compared with predictions. That perspective is well supported by experimental results; see Discussion for a review of evidence for negative prediction errors (e.g. pauses in dopaminergic firing) caused by negative experiences.

Such bursts of dopaminergic activity produce distinct changes in the synaptic weights of Go and No-Go neurons [11]. Several computational models have attempted to describe the learning process of the synapses of Go and No-Go neurons [12–15]. Among these models, the OpAL model provided simple and analytically tractable rules describing the changes in weights of Go and No-Go neurons as a function of outcome prediction errors [8]. However, no-one so far examined how the basal ganglia might estimate payoff and cost if they are both associated with the same action.

The goal of this paper is to show how the Go and No-Go neurons can learn the payoffs and costs of individual actions through local synaptic plasticity rules. We argue that the payoffs and costs of individual actions are not necessarily correlated (for instance, two actions might have comparable benefits, but very different costs), and strive to construct a model that is able to represent those independent dimensions of reinforcement for every single action. Ultimately, we confront the resulting model with experimental results.

Instead of constructing a new set of learning rules from scratch, we will employ the theory of striatal learning described in [16], which has been shown to account for diverse observations. That theory was originally developed to explain how the mean and the spread of the reinforcement signal could be learned by the basal ganglia network. In this article, we will prove that if the weights of Go and No-Go neurons change according to these rules, they can eventually represent payoff and cost. In summary, we show that a set of learning rules, originally constructed to estimate statistical properties of the reinforcement signal, can be reinterpreted as rules to estimate payoffs and costs. We thus extend both the interpretation of the striatal pathways of Collins and Frank and the striatal learning rules of Mikhael and Bogacz to ultimately obtain a consistent theory of learning the payoffs and costs of actions.

According to the experimental and modeling work mentioned above, dopaminergic activity encodes both information about motivational state and the outcome prediction error. However, if the dopaminergic neurons carried both signals, the striatal neurons would need a way to decode each signal and react appropriately, i.e. change their activity according to the motivation signal, and change the synaptic weights according to the prediction error. The prominent suggestion that motivation might be encoded in the average or tonic dopamine level, and outcome prediction errors in the burst or phasic activity [17] is hotly debated; it seems to be contradicted by the observation of fast-changing dopaminergic activity that encodes motivation [18–20]. Note, though, that these apparently divergent views could potentially be reconciled–see e.g. [21]. Anyhow, the motivation and teaching signals could both be provided by other means. For example, the activity of striatal cholinergic neurons may inform what the dopaminergic neurons encode at the moment [20]. In this paper, we assume that striatal neurons can read out both motivation and teaching signals encoded by dopaminergic neurons, and we leave the details of the mechanisms by which they can be distinguished to future work.

## Results

Inspired by the OpAL model [8], we assume that synaptic weights within the Go pathway encode positive consequences of actions, that is the positive reinforcement caused by food, drink or other appetitive stimuli obtained through actions. More precisely, we claim that the typical payoff of a particular action *a* in a particular situation *s* is encoded in the strength of the connections from the cortical neurons selective for the situation to the striatal Go neurons selective for the action. We denote these weights by *G*(*s*, *a*) (see Fig 1), and propose that after learning, the weights *G* represent the mean payoff for an action. Mathematically, the collective strength of the weights *G* corresponds to a single, non-negative number. The negative consequences, on the other hand, are encoded in the synaptic connections of striatal No-Go neurons. Negative consequences should be understood as the negative reinforcement induced by aversive stimuli such as pain, effort or disgust. We denote their weights by *N*(*s*, *a*), and propose that after learning, they represent the mean cost of an action. Just as with *G*, we mathematically represent the collective strength of the weights *N* by a single, non-negative number.

**Fig 2:**
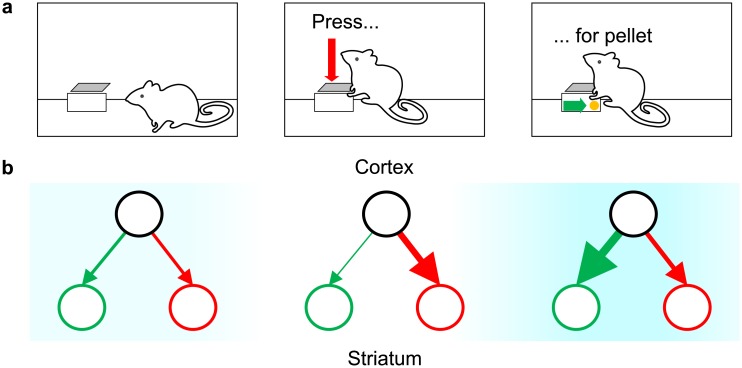
Qualitative description of learning payoffs and costs. (a) Operant conditioning chamber setup: a rat obtains a food pellet by pressing a lever. (b) Diagrams of changes in the weights *G* and *N* associated with lever-pressing at each stage of the experience presented in panel (a). In all diagrams, the black circles represent the cortical neurons selective for the state (being in the operant box), and the green and red circles represent the Go and No-Go populations of striatal neurons, respectively, selective for the action (pressing the lever). The thickness of the arrows linking the circles represents the connection strength between the respective neuron populations. The blue shading in the background indicates the strength of the immediate reinforcement, with a colour intensity proportional to the magnitude of reinforcement.

To learn the positive and negative consequences of actions respectively, the striatal neurons can take advantage of the fact that these consequences typically occur in different moments in time. Let us consider a situation in which an animal performs an action that involves an effort in order to obtain a reward: Fig 2a sketches a task in which a rat is given the opportunity to press a lever in order to obtain a food pellet. Due to the effort, the instantaneous reinforcement during the course of this action is negative at first, while pressing the lever. Then, it turns positive at the time the payoff is received. Fig 2b sketches the resulting changes in the synaptic weights. The leftmost display shows the initial weights. While making an effort to perform an action, the outcome prediction error is negative. Similarly as in previous models [8, 12], we assume that the negative prediction error results in a strengthening of *N* (compare the red arrows in the middle and the left displays in Fig 2b). This allows the weights *N* to encode negative consequences. Later, reception of the payoff causes a positive prediction error, which strengthens *G*. This leads the weights *G* to encode the positive consequences. Here, we assumed that–at baseline dopamine level–positive prediction errors trigger more plasticity in the Go pathway than in the No-Go pathway, while negative prediction errors affect the No-Go pathway more than the Go pathway. In Discussion, we will review data suggesting that the properties of D1 and D2 receptors allow this assumption. Generally, if an experience involves both positive and negative consequences, both weights are increased during the experience (compare the right and the left displays in Fig 2b).

To mathematically implement these ideas, we need to model the weighs of the Go pathway *G*(*s*, *a*), the weighs of the No-Go pathway *N*(*s*, *a*), and the prediction error. The outcome prediction error, which we denote by *δ*, quantifies the difference between the expected reinforcement and the received reinforcement *r* after executing action *a* in situation *s*. If *r* is negative, we shall speak of cost, and when *r* is positive, we shall speak of payoff or reward. The expected reinforcement, on the other hand, directly corresponds to the expected payoffs and costs, which–according to our theory–are represented by the synaptic weights *G* and *N*. We take the expected reinforcement to be the average over the expected payoff and the expected cost. Altogether, we model the outcome prediction error as

$$\begin{array}{c} \delta =r-\frac{1}{2}(G(s,a)-N(s,a)). \end{array}$$ (1)

It should be clarified that this definition of the prediction error differs from one in the original model [16], in that we introduced here a factor 1/2. This factor allows *G* and *N* to converge to the exact payoffs and cost, and not to values proportional payoffs and costs, and hence increases the clarity of the exposition. However, since value cannot be measured directly, the overall scaling of values through this factor is not observable, but a mere convention.

**Fig 3:**
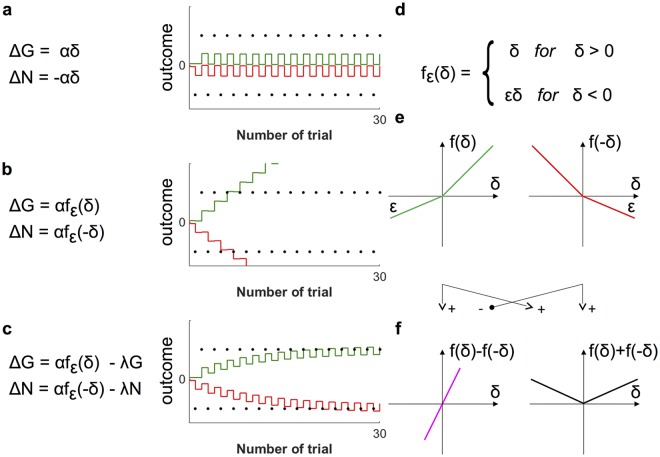
The incremental construction of the learning rules. (a)–(c) The different stages in the construction of the learning rules. All panels feature a mathematical formulation of the rules at the given stage and a simulation of these rules. The reinforcements in those simulations, indicated by black dots, alternate between a fixed payoff of magnitude 20 and a fixed cost of −20. The Go weights *G* are depicted in green, the negative No-Go weights −*N* are depicted in red. The parameters used in the simulations were *α* = 0.300, *ϵ* = 0.443 and λ = 0.093. (d)–(f) Definition, visualization and properties of the nonlinear function *f*_*ϵ*_.

Equipped with the quantities *δ*, *G* and *N*, we can formulate our theory of learning payoff and cost. To present the theory, we simply describe how the collective connection strengths *G*(*s*, *a*) and *N*(*s*, *a*) change when a prediction error *δ* is received after executing action *a* in situation *s*; we use Δ*G*(*s*, *a*) and Δ*N*(*s*, *a*) to denote the changes in relevant connection strengths. Note that any update only applies if the resulting weights are still positive—if an update would render a weight negative, that weight is set to zero instead. In all other cases, we follow Mikhael and Bogacz [16] in prescribing

$$\begin{array}{c} \Delta G(s,a)=\alpha f_{\epsilon }(\delta )-\lambda G(s,a) \end{array}$$ (2)

$$\begin{array}{c} \Delta N(s,a)=\alpha f_{\epsilon }(-\delta )-\lambda N(s,a), \end{array}$$ (3)

where *α* is the learning rate, *ϵ* is the slope parameter and λ the decay rate. The slope parameter *ϵ* controls the strength of the nonlinearity exhibited by the function *f*_*ϵ*_, which we introduce in Fig 3d and 3e. The nonlinearity of the function *f*_*ϵ*_ accounts for the fact that positive and negative prediction errors affect the weights differently. From here on, we drop the dependency of *G* and *N* on *a* and *s* to simplify notation. The dependency is still implicitly assumed unless stated otherwise.

There is a normative intuition for each term in the rules Eqs 2 and 3. These intuitions are most easily gained by following through a couple of steps to reconstruct the rules from scratch. We may start by observing that several models of learning in Go and No-Go neurons assume the effect of the prediction error on *G* to be opposite to its effect on *N* [7, 8]. We thus propose that Δ*G* and Δ*N* might simply be proportional to the prediction error and its negative, respectively. To see whether this proposal works, we formulate it mathematically and simulate the learning of an alternating sequence of costs −*n* and payoffs *p*. Fig 3a shows both the mathematical formulation and the simulation. There is a problem: the strengthening of *N* due to negative prediction error, caused by the cost, is always immediately reversed by the following positive prediction error caused by the payoff. The same is true for the changes in *G*. As illustrated by the simulation, there is no net effect of learning.

To overcome this problem, we proceed by damping the impact of negative prediction errors (which are usually caused by costs) on *G*, and the impact of positive prediction errors on *N*, by introducing a nonlinear transformation of the prediction errors. This transformation is given in form of a piecewise-linear function *f*_*ϵ*_, defined and depicted in panels d and e of Fig 3. The transformation leaves positive prediction errors invariant (*f*_*ϵ*_ (*δ*) is just the identity for *δ* > 0) but reduces the impact of negative prediction errors by scaling them down (for *δ* < 0, *f*_*ϵ*_ (*δ*) is linear with slope *ϵ* < 1). Hence, *f*_*ϵ*_ introduces a pathway-specific imbalance between learning from positive prediction errors and learning from negative prediction errors (which, as we point in Discussion, is in accordance with the properties of dopaminergic receptors on these pathways). For the construction at hand, it is also logical, since costs should not alter the estimate *G* of the payoffs and vice versa. Such damping can be achieved by replacing the simple proportionality to *δ* in the first proposal by a nonlinear dependence, mediated by the functions depicted in Fig 3e. We update our mathematical formulation accordingly, and again simulate the effects of the previously used reinforcement sequence—both these steps are illustrated in Fig 3b. The simulation shows that, while producing the appropriate tendencies, these rules cause unconstrained, ongoing strengthening of both connections. Such dynamics are neither biologically plausible nor useful to infer the actual payoff and cost.

Finally, to stop unconstrained strengthening and stabilize the weighs, we balance growth with decay. Adding decay terms to the mathematical formulation of the rules yields their final form Eqs 2 and 3. The simulation in Fig 3c suggests that the construction was successful: the final version of the rules allows the weights to converge to *p* and *n* respectively.

### Mathematical analysis

After providing an intuitive understanding of the learning rules and their mathematical formulation, we proceed to a more rigorous analytical treatment. We saw the potential of Mikhael and Bogacz’ [16] rules to learn payoffs and costs. Appropriate choice of parameters is key to unlock that potential, and we shall now investigate how that choice must be made. In particular, we will derive certain relations between parameters that must be satisfied for payoff and cost to be learned.

**Fig 4:**
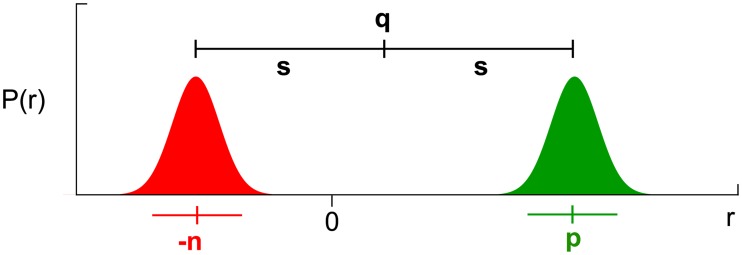
The relation of reinforcement statistics to payoff and cost. The graph shows a representative reinforcement distribution over the magnitude *r* of all received reinforcements. The parts of the distribution that indicate negative reinforcements (costs) are colored red, while the parts that indicate positive reinforcements (payoffs) are colored green. The mean *q* and the mean spread *s* are indicated above the distribution, the mean cost −*n* and the mean payoff *p* are indicated below the distribution.

Originally, the rules Eqs 2 and 3 were meant to describe learning of reinforcements statistics. Mikhael and Bogacz [16] showed that after learning, particular combinations of *G* and *N* will encode the mean ER and the mean spread E|R−ER| of the received reinforcements. For further reference, we denote these important statistics by q≔ER and s≔E|R−q|. How are the mean and the mean spread of received reinforcements related to payoff and cost? Consider the reinforcement statistics of an action that reliably requires effort (corresponding to negative reinforcement) to produce a payoff (which corresponds to positive reinforcement). Assume that these reinforcements are clearly negative and positive respectively, such that one will not be confused for the other even in the presence of noise. Repeat that action multiple times, and record all received reinforcements, the costs as well as the payoffs. Finally, analyze how all these received reinforcements are distributed. If an effort was required to earn the payoff, the distribution of reinforcements will turn out bimodal, as schematically shown in Fig 4. It features two peaks, one centered around the mean payoff *p*, and one centered around the mean cost −*n*, respectively. Fig 4 also shows the mean *q* and the mean spread *s* of that distribution. We observe that payoffs and costs are both exactly one mean spread *s* away from the center *q* of the distribution—the payoff above, and the cost below. This implies that there is, at least in this representative case, a strong connection between payoffs and costs and the reinforcement statistics:

$$\begin{array}{c} p=q+s \end{array}$$ (4)

$$\begin{array}{c} -n=q-s \end{array}$$ (5)

This connection allows us to set up conditions for the result of learning: if *G* and *N* are to represent payoff and cost, they must approach *q* + *s* and −*q* + *s* respectively. Equivalently, we can ask for 1/2(*G* − *N*) and 1/2(*G* + *N*) to approach *q* and *s* in the course of learning.

After revealing the link between reinforcement statistics and payoff and cost, we are ready to derive the relations necessary to learn the latter. To that end, we first determine the connection strengths *G* and *N* that result from training on stochastic reinforcements. Such uncertain reinforcements are sampled at random from a fixed distribution. Then, we implement the newly identified conditions, demanding for 1/2(*G* − *N*) to approximate *q* and 1/2(*G* + *N*) to approximate *s* after training is finished. From these conditions, we will be able to derive the desired parameter relations.

Working through these steps is simpler after changing variables from *G* and *N* to *Q* ≔ 1/2(*G* − *N*) and *S* ≔ 1/2(*G* + *N*) right away. We saw that the new variables *Q* and *S* have a clear computational interpretation: if learning goes as planned, *Q* and *S* track the mean *q* and the mean spread *s* of the experienced reinforcement. To determine how *Q* and *S* change due to prediction errors *δ*, we simply add and subtract the update rules Eqs 2 and 3. Certain convenient properties of the nonlinear functions *f*_*ϵ*_ help to further simplify the resulting equations: Fig 3f shows that subtracting and adding functions depicted in Fig 3e give functions proportional to identity and absolute value, respectively. Explicitly, *f*_*ϵ*_(*x*) − *f*_*ϵ*_(−*x*) = (1 + *ϵ*)*x* and *f*_*ϵ*_(*x*) + *f*_*ϵ*_(−*x*) = (1 − *ϵ*) |*x*|. Exploiting these properties, we obtain

$$\begin{array}{c} \Delta Q=\alpha_{Q}\delta -\lambda Q \end{array}$$ (6)

$$\begin{array}{c} \Delta S=\alpha_{S}\left|\delta \right|-\lambda S. \end{array}$$ (7)

Here, for brevity of notation, we introduced the effective learning rates *α*_*Q*_ = *α*(1 + *ϵ*)/2 and *α*_*S*_ = *α*(1 − *ϵ*)/2. Note that the changes of *Q* and *S* are proportional either to the prediction error itself or to its absolute value, in contrast to the changes of *G* and *N*.

Now, let us determine the strengths of the weights *G* and *N*, or equivalently of the variables *Q* and *S*, after many encounters with an action. When learning the reinforcements of a previously unknown action, *Q* and *S* typically change a lot during the first trials. These changes then get smaller and smaller as more experience is integrated—the learning curve plateaus. After enough trials, *Q* and *S* stop changing systematically, and start to merely fluctuate about some constant values, which we denote by Q* and S* and refer to as equilibrium points. In mathematical terms, directed learning stops when we may expect *Q* and *S* to remain unchanged by another trial, i.e. when E(ΔQ)=E(ΔS)=0. If that stage is reached, the equilibrium points can be inferred by computing the mean value of the fluctuating variables: $Q^{*}=EQ$ and $S^{*}=ES$. With these identities and the learning rules Eqs 6 and 7, we can determine the equilibrium points Q* and S*:

$$\begin{array}{c} 0=E\;\Delta Q=E\;[\alpha_{Q}(R-Q)-\lambda Q]=\alpha_{Q}(q-Q^{*})-\lambda Q^{*} \end{array}$$ (8)

$$\begin{array}{c} 0=E\;\Delta S=E\;[\alpha_{S}|R-Q|-\lambda S]=\alpha_{S}E\;|R-Q|-\lambda S^{*}. \end{array}$$ (9)

To solve these equations, we shall make the additional assumption that the fluctuations of *Q* about Q* are small. This assumption is justified whenever *α* is sufficiently small, and allows us to approximate $E\;|R-Q|\approx E\;|R-Q^{*}|$. Collecting all those intermediate results, we may solve Eqs 8 and 9 for the equilibrium points. The solutions read

$$\begin{array}{c} Q^{*}=c_{Q}\;q \end{array}$$ (10)

$$\begin{array}{c} S^{*}\approx c_{S}\;E|R-c_{Q}q|, \end{array}$$ (11)

with *c*_*Q*_ = *α*_*Q*_/(*α*_*Q*_ + λ) and *c*_*S*_ = *α*_*S*_/λ. Those are the approximate values of *Q* and *S* after learning.

Next, we need to implement the conditions we inferred from Fig 4. Thanks to our choice of variables, this simply amounts to requiring that *Q* converge to the mean reinforcement *q*, and *S* to the mean spread *s*, i.e. requiring Q* = *q* and S* = *s*. Inserting the approximate values from Eqs 10 and 11 produced by the learning rules, we obtain

$$\begin{array}{c} c_{Q}\;q=q \end{array}$$ (12)

$$\begin{array}{c} c_{S}\;E|R-c_{Q}q|=s \end{array}$$ (13)

These equations are central to this publication. Their left-hand side represents the result of learning according to Mikhael and Bogacz’ [16] rules. Their right-hand side specifies what needs to be learned if *G* and *N* really represented payoffs and costs, as Collins and Frank hypothesized [8]. Equating the left-hand and the right-hand side amounts to merging both theories. It allows us to determine how the parameters would be related if both theories were exactly true: for Eqs 12 and 13 to hold, *α*, λ and *ϵ* must take values such that *c*_*Q*_ = 1 and *c*_*S*_ = 1.

This result evokes several questions: Is it at all possible to satisfy the derived conditions? What do the conditions mean with respect to the parameters *α*, λ and *ϵ*? And finally, is there a practical way to determine sets of parameters *α*, λ and *ϵ* which—at least approximately—satisfy the conditions? We discuss each of these questions in the following paragraphs.

Firstly, is it possible to satisfy *c*_*Q*_ = 1 and *c*_*S*_ = 1 exactly? Examining the definition *c*_*Q*_ = *α*_*Q*_/(*α*_*Q*_ + λ) quickly reveals that letting *c*_*Q*_ → 1 would amount to letting λ → 0. To see why this is the case, consider that *c*_*Q*_ → 1 amounts to λ/*α*_*Q*_ → 0. However, *α*_*Q*_ is an effective learning rate, and so must take values smaller then one. Thus, we really need to let λ → 0. Now, we derived above that after learning, *S* will fluctuate about its equilibrium point $S^{*}\approx c_{S}\;E|R-c_{Q}q|$ with *c*_*S*_ = *α*_*S*_/λ. In order to keep the equilibrium point S* finite as λ → 0, we would therefore be forced to have *α*_*S*_ → 0 also. This, though, would pose a real problem: *α*_*S*_ is the effective learning rate for *S*—having it vanish would imply stopping learning in *S* all together. We must conclude that strict satisfaction of the constraints *c*_*Q*_ = 1 and *c*_*S*_ = 1 is not compatible with non-vanishing learning rates that lead to a finite equilibrium. Specifically, *c*_*Q*_ = 1 can only ever hold approximately if the spread *s* is to be learned in finite time. Nevertheless, no such problem arises when *c*_*S*_ is set to 1 exactly.

Now, what do the constraints *c*_*Q*_ ≈ 1 and *c*_*S*_ = 1 mean in terms of the parameters *α*, λ and *ϵ*? In the previous paragraph, we saw that *c*_*Q*_ ≈ 1 is equivalent to λ/*α*_*Q*_ ≈ 0. Since both λ (a decay constant) and *α*_*Q*_ (an effective learning rate) are inherently positive, we may rewrite this as λ/*α*_*Q*_ ≪ 1. Inserting the definition *α*_*Q*_ = *α*(1 + *ϵ*)/2 immediately yields

$$\begin{array}{c} 2\lambda \ll \alpha (1+\epsilon ) \end{array}$$ (14)

The other condition, *c*_*S*_ = 1, is easily translated analogously. We need only use the definitions *c*_*S*_ = *α*_*S*_/λ and *α*_*S*_ = *α*(1 − *ϵ*)/2 to obtain

$$\begin{array}{c} 2\lambda =\alpha (1-\epsilon ). \end{array}$$ (15)

Eqs 14 and 15 constitute the exact relations between the parameters *α*, λ and *ϵ* that need to hold for payoffs and costs to be estimated accurately. They cannot be further simplified, but we may use them to gain some more insight into the required magnitudes of the individual parameters: by substituting 2λ according to Eq 15 on the right-hand side of Eq 14, one obtains a condition of the form 1 − *ϵ* ≪ 1 + *ϵ*. Now, given that the intended range for *ϵ* is [0, 1], one quickly reaches the conclusion that *ϵ* ≈ 1. Reinserting this into Eq 14 yields λ ≪ *α*. In conclusion, we found that it is necessary (though not sufficient) for accurate learning of payoffs and costs to maintain a small, but non vanishing nonlinearity *ϵ* in the transmission of the prediction error signal, as well as a non vanishing decay rate λ, which is much smaller than the learning rate *α*.

Finally, how can such parameters *α*, λ and *ϵ* practically be determined? To implement the conditions *c*_*Q*_ ≈ 1 and *c*_*S*_ = 1, one can for instance express λ and *ϵ* in terms of *α*, *c*_*Q*_ and *c*_*S*_. It is tedious, but without conceptual difficulty to invert the definitions of *c*_*Q*_ and *c*_*S*_ in order to yield *ϵ* = (1 − *c*_*S*_(1/*c*_*Q*_ − 1))/(1 + *c*_*S*_(1/*c*_*Q*_ − 1)) and λ = *α*(1 − *ϵ*)/(2*c*_*S*_). Then, one chooses *α* freely at one’s convenience, and *c*_*Q*_ and *c*_*S*_ close (or, in case of *c*_*S*_, equal) to one. Importantly, *c*_*Q*_ must be chosen smaller then one to result in a positive λ. From these choices, one finally obtains *ϵ* and λ to work with the chosen *α*. Our simulations suggest that even values such as *c*_*Q*_ = 0.7 and *c*_*S*_ = 0.9, in combination with a learning rate of, say *α* = 0.3, are close enough to one to allow reasonably accurate estimations of payoff and cost. This can be seen in Fig 3: the simulations shown in there used those exact settings, which equivalently means that *ϵ* = 0.443 and λ = 0.093.

In summary, we used a statistical argument–the connection between payoffs and costs and the reinforcement statistics–to determine conditions under which payoffs and costs can be learned with the update rules Eqs 2 and 3.

### Deterministic reinforcement sequences

In the preceding section, we derived relations that are necessary for successful learning of payoff and cost. If rewards are awarded stochastically, those relations are also sufficient for successful learning. But what happens to the weighs *G* and *N* if the received reinforcements follow a strong pattern? Assume, for instance, that an action reliably yields a fixed cost −*n* followed by a fixed payoff *p*. Under which additional conditions do *G* and *N* then still reflect the magnitudes of payoff and cost after learning?

To answer that question, we must again determine the connection strengths that result from experiencing the action time and again. Now, we do not have to rely on a probabilistic treatment—when the pattern of the reinforcements is fully known, it is possible to determine the evolution of *G* and *N* exactly. As in the previous section, we will concentrate on the result of learning rather than on its dynamics. Here, this amounts to determine the fixed points of the learning rules. These fixed points are simply those values of *G* and *N* (or equivalently of the alternative variables *Q* and *S* we defined above) that are invariant under the updates caused by the action. We denote the fixed points by G* and N*, or Q* and S*. During learning, the variables converge to their respective fixed points and cease to change notably once they arrive in their vicinity.

First, we focus on determining the fixed point of *Q*. Note that each encounter with the action yields two updates of *Q*: one due to the cost and one due to the payoff. Mathematically, we can formulate this as

$$\begin{array}{c} Q_{\text{after}\;\text{action}}=Q_{\text{before}\;\text{action}}+{\left(\Delta Q\right)}_{\text{cost}}+{\left(\Delta Q\right)}_{\text{payoff}}. \end{array}$$ (16)

To find Q*, demand that these successive updates have no net effect on *Q*: If *Q*_after action_ equals *Q*_before action_, then *Q*_before action_ can rightfully be called fixed point. If this is so, the two updates must have canceled each other:

$$\begin{array}{c} {\left(\Delta Q\right)}_{\text{cost}}+{\left(\Delta Q\right)}_{\text{payoff}}=0 \end{array}$$ (17)

This condition, in combination with the update rules Eqs 2 and 3, allows to determine Q* in terms of *p*, *n* and the parameters *α*, *ϵ* and λ. First, we use the update rule Eq 6 for *Q* to write (Δ*Q*)_*cost*_ as

$$\begin{array}{cc} {\left(\Delta Q\right)}_{\text{cost}} & =\alpha_{Q}(r_{\text{cost}}-Q_{\text{before}\;\text{action}})-\lambda Q_{\text{before}\;\text{action}}\\ & =\alpha_{Q}(-n-Q_{\text{before}\;\text{action}})-\lambda Q_{\text{before}\;\text{action}}. \end{array}$$

Then, one uses the rule again to write (Δ*Q*)_payoff_ as

$$\begin{array}{cc} {\left(\Delta Q\right)}_{\text{payoff}} & =\alpha_{Q}(r_{\text{payoff}}-Q_{\text{after}\;\text{cost}})-\lambda Q_{\text{after}\;\text{cost}}\\ & =\alpha_{Q}(p-Q_{\text{after}\;\text{cost}})-\lambda Q_{\text{after}\;\text{cost}}\\ & =\alpha_{Q}(p-(Q_{\text{before}\;\text{action}}+{\left(\Delta Q\right)}_{\text{cost}}))-\lambda (Q_{\text{before}\;\text{action}}+{\left(\Delta Q\right)}_{\text{cost}}). \end{array}$$

Finally, one substitutes (Δ*Q*)_cost_ from above into this expression, and then inserts (Δ*Q*)_cost_ and (Δ*Q*)_payoff_ into Eq 17. Solving the equation for *Q*_before action_, which in case of Eq 17 is identical to Q*, yields

$$\begin{array}{c} Q^{*}=\frac{1}{2-\alpha_{Q}-\lambda }(n(\alpha_{Q}+\lambda -1)+p), \end{array}$$ (18)

where *α*_*Q*_ = *α*(1 + *ϵ*)/2. Now, recall that the definition of *Q* in terms of *G* and *N* is *Q* = 1/2(*G* − *N*), and that true payoffs and costs of in this model are *p* and *n*. If *G* and *N* represented the true payoffs and costs after learning, it must be true that G* ≈ *p* and N* ≈ *n*, and thereby

$$\begin{array}{c} \frac{1}{2-\alpha_{Q}-\lambda }(n(\alpha_{Q}+\lambda -1)+p)\approx \frac{1}{2}(p-n). \end{array}$$ (19)

Just as Eqs 12 and 13, this equation is an interface between the results of Mikhael and Bogacz’ [16] update rules on the left-hand side and the requirement that Go and No-Go weights encode payoffs and costs on the right-hand side. For both sides to agree, we must have

$$\begin{array}{c} \alpha_{Q}+\lambda \approx 0. \end{array}$$ (20)

This is a novel condition for learning the correct magnitudes of payoffs and costs from a deterministic reinforcement pattern. The definition of *α*_*Q*_ and the previously derived conditions in Eqs 14 and 15 may be used to transform this novel condition into the simpler form *α* ≪ 1.

Next, we repeat the same analysis for *S*. Since we search for additional conditions on the parameters, we are free to use the original conditions in Eqs 14 and 15 to simplify our calculations. The only complication we encounter is the appearance of *Q* in the update rules of *S*, which we resolve by substituting *Q* with Q*, acknowledging that the fixed points of *S* and *Q* depend on each other. We arrive at

$$\begin{array}{c} S^{*}\approx \frac{1}{2}(p+n). \end{array}$$ (21)

Again, using the definition *S* = 1/2(*G* + *N*) allows comparing the result of learning with the strengths required to represent payoffs and costs. We immediately find that G* ≈ *p* and N* ≈ *n* already hold. Thus, Eq 20 is the only additional condition for successful learning of payoff and cost from reinforcements that follow a strong pattern.

From the results presented in this section, we conclude that the learning rules Eqs 2 and 3 facilitate learning of the magnitudes of fixed payoffs and costs that occur reliably one after the other. However, we also saw that this is only true if Eq 20 holds in addition to the conditions that we derived in the previous section.

### Summary of analytic results

The analysis above revealed the conditions under which the striatal plasticity rules Eqs 2 and 3 could learn the magnitudes of the payoffs and costs of actions. We identified the conditions in two different paradigms: first, we investigated learning from purely stochastic reinforcements sampled from a fixed distribution. Then, we considered a deterministic pattern of reinforcements. We obtained two key results:

- Consider a reinforcement distribution—obtained from multiple encounters with an action—that is shaped by payoffs and cost, as the one shown in Fig 4. If trained on reinforcements sampled from that distribution, the plasticity rules Eqs 2 and 3 will enable learning of the mean payoffs and costs if
  $$\begin{array}{c} 2\lambda \ll \alpha (1+\epsilon ) \end{array}$$ (22)
  $$\begin{array}{c} 2\lambda =\alpha (1-\epsilon ) \end{array}$$ (23)
  hold. These conditions imply–but do not follow from–a non-vanishing but small nonlinearity in the transmission of the prediction error, and a non-vanishing but small decay of the connection weights. Here, a small decay is characterized by a decay rate λ which is small compared to the learning rate *α*.
- If trained on a pattern of reinforcements that alternates between payoffs of magnitude *p* and costs of magnitude *n*, the plasticity rules Eqs 2 and 3 will capture those exact payoffs and costs if, in addition to Eqs 22 and 23,
  $$\begin{array}{c} \alpha \ll 1 \end{array}$$ (24)
  holds. In words, unbiased learning of payoffs and costs in deterministic scenarios explicitly requires a small learning rate *α*.

### Simulations of learning

The previous sections revealed what to expect from training the learning rules Eqs 2 and 3 on certain types of reinforcement. Specifically, we investigated the connection strengths *G* and *N* after many experiences of either totally predictable or totally random reinforcements. In this section, we aim to confirm and extend those results using numerical simulations rather than analytic methods.

**Fig 5:**
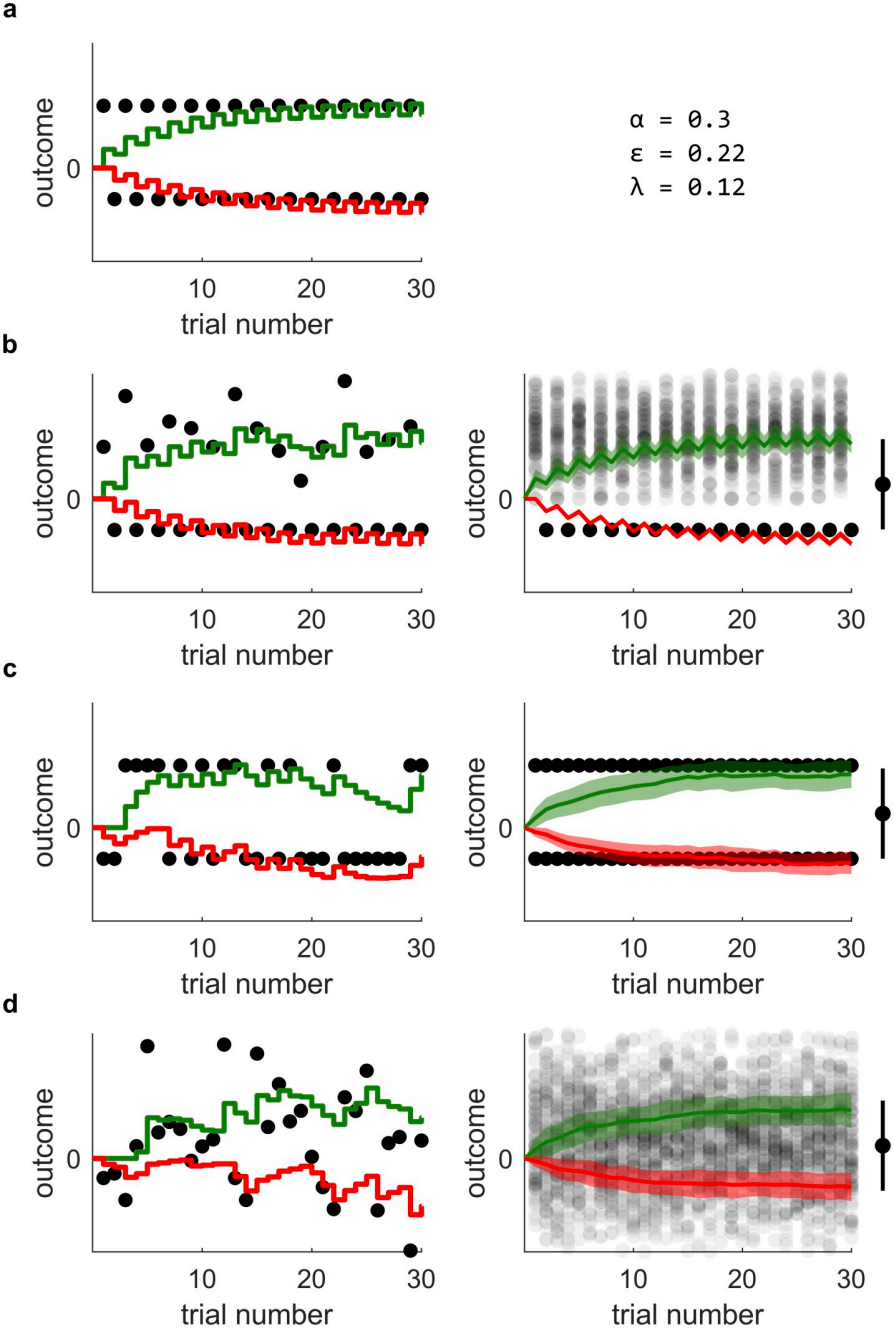
Simulations of learning. In all graphs, the collective strength *G* of the Go weights is depicted in green, while the negative collective strength −*N* of the No-Go weights is depicted in red. The received reinforcements are indicated by solid black dots in the panels on the left, and by transparent black dots in the panels on the right. Each simulation shows how *G* and *N* change due to the reception of 30 prediction errors. Panel (a) contains a simulation based on predictable, alternating reinforcements. It also contains the parameter values used for the simulations. Panels (b) to (d) show both single and averaged simulations of stochastic reinforcements: On the left, we show a single sequence of learning, with reinforcements sampled from different distributions. On the right, we show averages over many such sequences of learning. There, the mean weights are depicted as green and red lines, while the shaded green and red areas around these lines of *G* and *N* in the right column indicate one standard deviation. The bars to the right of the averaged learning curves indicate the mean and mean spreads of the respective reinforcement distributions.

Fig 5 shows the results of simulating the gradual change of connection weights in four different tasks. In all those simulations, *G* and *N* change according to the learning rules Eqs 2 and 3. The parameters we used roughly fulfill the conditions Eqs 12 and 13 for learning of the correct magnitudes of payoffs and costs, but are also chosen to facilitate quick convergence. The values presented in Fig 5a mirror that compromise.

The simulation in Fig 5a is based on a repeating an action that reliably results in a cost −*n*, followed by a payoff *p*. An analytic treatment of that case can be found in the previous sections. Both weights constantly oscillate due to the alternation of payoff and costs. This oscillating behavior is superimposed with learning curves that take the weights from their initial values towards the magnitudes of the payoffs and costs respectively. After 30 trials, *G* and *N* represent good approximations of *p* and *n*. Fig 5b is similar to Fig 5a, with a slight variation: Just as in Fig 5a, payoffs and costs alternate reliably. But while the cost is again held constant at −*n*, this time the payoff *P* is sampled from a fixed distribution (a normal distribution with mean *p* and non-vanishing variance) in each trial. Thus, the task includes both stochastic and deterministic components: each repetition of an action results in a fixed cost, which is followed by an uncertain reinforcement. The depicted simulations show that under such conditions, *N* eventually represents the cost *n*, while *G* converges towards the mean payoff p=EP.

Finally, Fig 5c and 5d contain simulations of repeated actions with reinforcements drawn completely at random from fixed distributions. In Fig 5c, the obtained reinforcements are valued either *p* or −*n*, with probabilities 1/2 each. In Fig 5d, reinforcements are sampled from a normal distribution with mean *μ*_*r*_ = 1/2(*p* − *n*) and standard deviation of $\sigma_{r}=1/2\sqrt{\pi /2}\left(p+n\right)$. We simulate the experience resulting from such actions by sampling reinforcements from a fixed distribution on each trial. The stochastic nature of this procedure causes the evolution of the weights *G* and *N* to be different each time the simulation is run. To overcome that effect and segregate random fluctuations from reproducible effects, we collect and average a large number of runs. Each row in Fig 5b–5d contains both a single run of the simulation and an average of 500 successive runs. In the above sections, we proved that in purely stochastic tasks, the weights would approximate key statistics of the reinforcement distribution after convergence. Those statistics are indeed approximated in the simulations, confirming the results of the analytic treatment above.

### Simulations of the effect of D2 blocking

In the previous sections, we focused on the change of the synaptic weights associated with a single action during the accumulation of experience. In this section, we redirect our attention. Instead of considering one action during learning, we now consider multiple actions after learning, and ask: can effects of dopamine depletion on choice behavior be explained in terms of payoffs versus costs?

**Fig 6:**
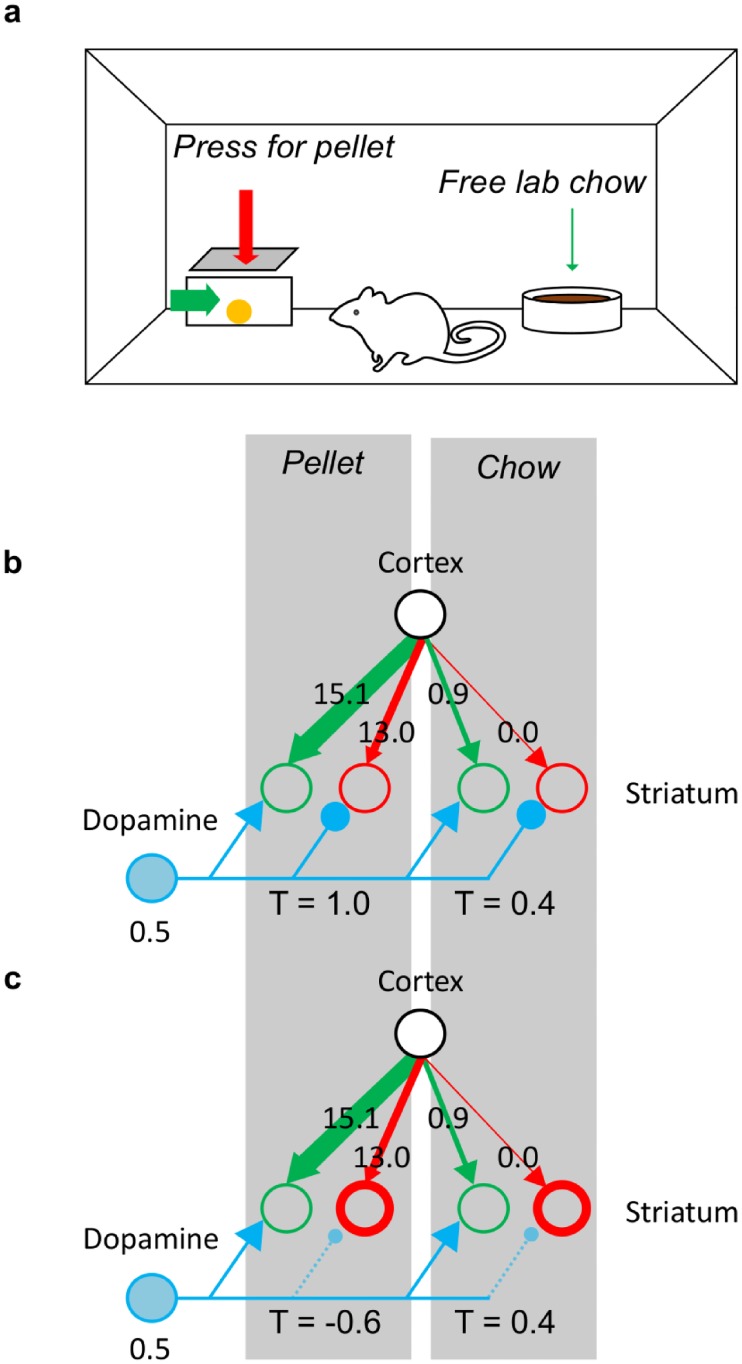
Effects of D2 blocking on the willingness to exert effort. (a) Schematic illustration of the experimental setup. (b) Action selection in control state. Green and red circles on the left denote striatal Go and No-Go neurons associated with pressing the lever, while the green and red circles on the right denote the neurons associated with approaching the free food. The strengths of the synaptic connections, which result from simulated learning, are indicated by the thickness of the arrows, and the labels. The parameters used for the simulations were obtained through a fit of the model to the experimental data. The blue circle represents a population of dopaminergic neurons, and its shading indicates the level of activity. (c) Action selection in the dopamine-depleted state. The notation is the same as in panel (b), with the thick red circles indicating enhanced activity in the No-Go population, which results from blocked dopaminergic inhibition (symbolised by the smaller inhibitory projections of the dopamine neurons).

In a classic experiment illustrated in Fig 6a, rats were given a choice between pressing a lever in order to obtain a nutritious pellet and freely available lab chow [22]. Normal animals were willing to work for pellets, but after blocking D2 receptors with the drug haloperidol they were not any more willing to make an effort and preferred a less valuable but free option. Collins and Frank [8] provided a mechanical explanation for this surprising effect. The theory proposed in this paper accounts for it in a conceptually similar but slightly simpler way. Here, we explain our modeling of the experiment and then describe the simulations—the differences to the account of OpAL model are pointed out in Discussion.

To model the experiment, we need to specify how the striatal weights *G* and *N* and the motivation signal transmitted by dopamine affect the output of the basal ganglia system, and how that output then affects choice. We refer to the output of the basal ganglia as the thalamic activity, denoted by *T*. *T* depends on the cortico-striatal weights *G* and *N*, and dopaminergic motivation signal denoted by *D*. Even though this relationship might admittedly be complex, we restrict ourselves to just capture the signs of the dependencies by using a linear approximation:

$$\begin{array}{c} T=DG-(1-D)N \end{array}$$ (25)

**Fig 7:**
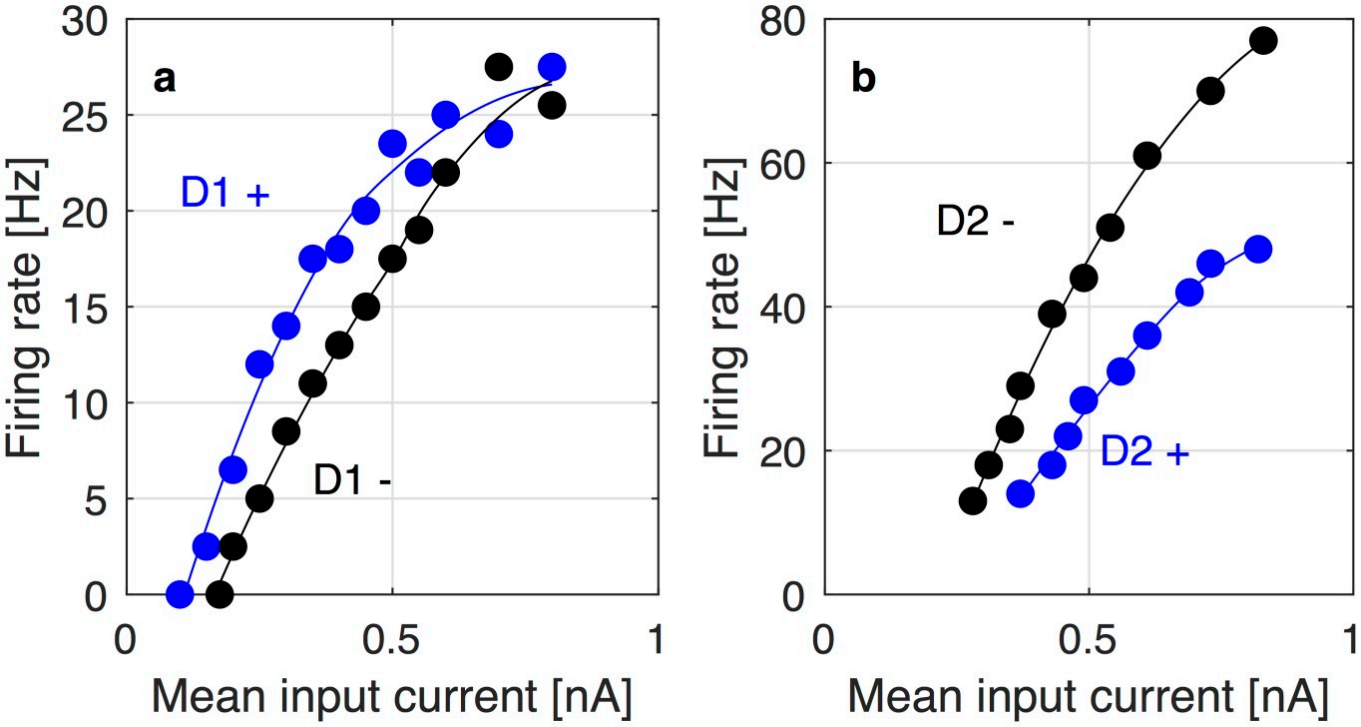
fI-curves of D1- and D2-expressing neurons at different levels of receptor activation. (a) fI-curves of a D1-expressing pyramidal neuron, replotted from [23]. The blue points are recorded from a neuron at a higher level of D1 receptor activation (e.g. with dopamine present), the black points are recorded at a lower level of receptor activation (e.g. without dopamine). Smooth curves have been obtained from the data through LOESS regression to serve as visual guides (black and blue lines). (b) fI-curves of a D2-expressing neuron, replotted from [24]. The blue points are recorded from a neuron at a higher level of D2 receptor activation (e.g. in the presence of the D2 agonist quinpirole), the black points are recorded from a neuron in the control group at a lower level of D2 activation (e.g. in the absence of the agonist). As in panel (a), LOESS curves (black and blue lines) have been added as visual guides.

In the above equation, the first term *DG* corresponds to the input from the striatal Go neurons. This term is positive because the projection from striatal Go neurons to the thalamus involves double inhibitory connections (see Fig 1) resulting in an overall excitatory effect. The activity of the Go neurons depends on synaptic weights *G*. We assume that their gain is modulated by the dopaminergic input *D*, extrapolated from the observation of an increased slope of the firing-input relationship in the presence of dopamine in pyramidal neurons expressing D1 receptors [23]. These data are replotted in Fig 7a. The second term −(1 − *D*)*N* corresponds to the input from the striatal No-Go neurons. It has a negative sign because the projection form the No-Go neurons to the thalamus includes three inhibitory connections. The activity of the striatal No-Go neurons depends on their synaptic weights *N*, and we assume that their gain is reduced by dopamine, so the synaptic input is scaled by (1 − *D*). This assumption is based on data showing that agonists reduce the slope of the firing-input relationship of striatal No-Go neurons [24], which are replotted in Fig 7b. Those assumptions about the impact of dopamine on the activity of striatal neurons are backed up by detailed modeling studies [25, 26], which predict precisely that dopamine enhances activity in the Go- and inhibits activity in the No-Go pathway. In Eq 25, we further assume that *D* ∈ [0, 1] and that the value of *D* = 0.5 corresponds to a baseline level of dopamine for which both striatal populations equally affect the thalamic activity.

Although arising from a slightly different induction, the action value defined by Eq 25 is directly proportional to the action value proposed by Collins and Frank, which is defined by Eq 4 of their publication [8]: *Q* ∝ *β*_*G*_*G* − *β*_*N*_*N*. One easily verifies the direct proportionality of the two expressions by rewriting

$$\begin{array}{c} D=1/2(1+(\beta_{G}-\beta_{N})/(\beta_{G}+\beta_{N})). \end{array}$$

How does thalamic activity affect choice? Again, we use a very simple dependency to capture the key aspects of that relationship: In our model of the experiment, we calculate the thalamic activity for each option. Then, we add some random noise independently to each option. Finally, all options with negative noisy thalamic activity are discarded, and the option with the highest noisy thalamic activity is chosen. If the noisy thalamic activity is negative for all available options, no choice will be made; the model defaults to staying inactive.

Often in similar situations, the softmax rule is the preferred choice procedure. According to that rule, one should first transform the set of different action values (or thalamic activities in this case) into a probability distribution over the available actions, by use of the softmax function. Then, one should sample an action from that distribution, and declare it the choice of that trial. Collins and Frank’s OpAL model [8] exemplifies the use of the softmax rule.

**Fig 8:**
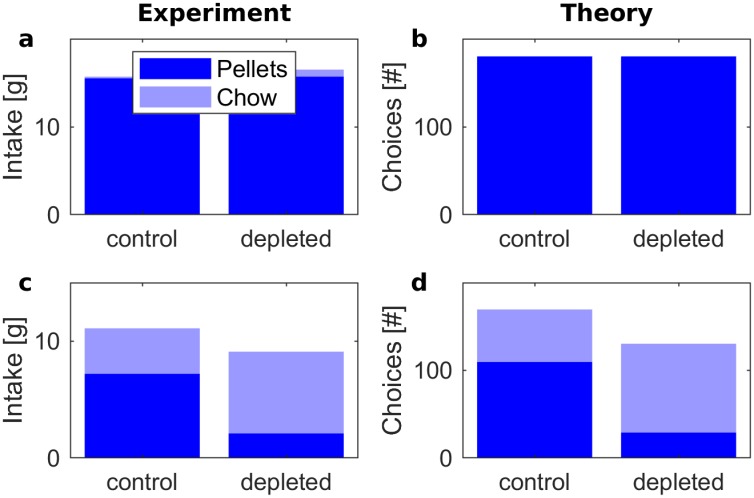
Frequency of choosing pellets (dark blue) and lab chow (light blue) in control and D2-blocked states. The top displays (a) and (b) correspond to a condition with free pellets, while the bottom displays (c) and (d) correspond to a condition where pressing a lever was required to obtain a pellet. The left displays (a) and (c) re-plot experimental data. The values in (a) were taken from Figure 1 in the paper by Salamone et al. [22]: pellet consumption was 15.5g and 15.7g in control and D2-blocked state, while chow consumption was 0.2g and 0.8g respectively. The values in (c) were taken from Figure 4 in [22]: pellet consumption was 7.2g and 2.1g in control and D2-blocked state, while chow consumption was 3.9g and 7g respectively. The right displays (b) and (d) show the results of simulations. The parameters used to simulate learning were *α* = 0.1, *ϵ* = 0.6327 and λ = 0.0204.

We deliberately decided against this conventional approach and in favor of the above-described procedure to accommodate a certain feature of the data presented in [22]: The group with D2 antagonist differed from the control group not only in their willingness to work for food but also in their overall food consumption. The rats with D2 antagonist consumed less food in total (see Fig 8c). We can hope to capture this effect with our model, since it allows for the possibility to make no choice at all, and thus consume neither of the food items. A softmax decision rule, on the other hand, forces a choice on each trial, and must therefore always lead to the same number of consumed food items.

Finally, how does the drug haloperidol affect the thalamic activity, and hence choice? Haloperidol is a D2 antagonist; it blocks the D2 receptors on the medial spiny neurons of the No-Go pathway. This blocking reduces the (inhibiting) impact of dopamine on the activity *N* of that pathway. To account for this in our model, we introduce another factor *κ*_*N*_ ∈ [0, 1] into our expression for the thalamic activity:

$$\begin{array}{c} T=DG-((1-\kappa_{N}D)N. \end{array}$$ (26)

The parameter *κ*_*N*_ controls the how much dopamine affects the activity of the No-Go pathway *N*, and is hence suitable to model D2-blocking: *κ*_*N*_ = 1 recovers the normal thalamic activity given in Eq 25, while *κ*_*N*_ = 0 (total blocking) fully removes the impact of dopamine on the indirect pathway, leading to completely uninhibited activity *N*. In the control group of the experiment, *κ*_*N*_ is set to 1 (no medication is administered, no blocking happens). In the group that received the medication, *κ*_*N*_ is a free parameter that must be fitted to the data. The best fit featured *κ*_*N*_ = 0.7507, corresponding to blocking of D2 receptors with an efficiency of roughly 25%.

Fig 6b illustrates how the model can account for the behaviour when the dopamine level has a normal baseline value. In the figure, the strength of the cortico-striatal connections is denoted by the labels and the thickness of arrows. Pressing the lever gives a high payoff, so the weights of Go neurons selective for this action are strong, but it also has a substantial cost, so the No-Go weights are also present. On the other hand, the free food is not particularly nutritious so the Go weights are weak, and there is no cost, so the No-Go weight is negligible. When no medication is administered, the positive and negative consequences are weighted equally, so the thalamic neurons selective for pressing the lever have overall slightly higher activity, which ultimately leads to a higher likelihood for this action to be chosen over the free option. By contrast, Fig 6c shows that when the D2 receptors are blocked, costs are weighted more than payoffs, and the thalamic activity associated with pressing the lever decreases. Approaching free food has only negligible cost; therefore, the activity of thalamic neurons selective for this option is now higher, and this action is overall more likely to be chosen.

A quantitative fit of our model to Salamone et al.’s experimental results [22] is illustrated in Fig 8. The panels on the left side in Fig 8 summarize experimental data: the top-left display corresponds to a condition in which both high-valued pellets and the low-valued lab chow were freely available. In this case, the animals preferred pellets irrespective of the dopamine level. The bottom-left panel corresponds to the condition in which the animal had to press a lever in order to obtain a pellet, and as mentioned before, after injections of a D2 antagonist they started to prefer the lab chow.

In our model of the experiment, we run through a sequence of trials mimicking those illustrated in Fig 6: on each trial, the model makes a choice between two actions—pressing a lever or approaching lab chow—or remains inactive. Before the main experiments, the animals were trained to press a lever to obtain rewards and were exposed to the lab chow [22]. To parallel this in simulations, the model was first trained such that it experienced each action a number of times, received corresponding payoffs and costs, and updated its weights according to Eqs 2 and 3. The weights resulting from that learning are reported in Fig 6b and 6c. Then, the model was tested with and without blocking, e.g. with *κ*_*N*_ a variable and *κ*_*N*_ fixed to one. As described in Materials and Methods, the parameters of the model were optimized to match experimentally observed behavior. As shown in the right displays in Fig 8, the model was able to reproduce the observed pattern of behavior. This illustrates the model’s ability to capture both learning about payoffs and costs associated with individual actions and the effects of the dopamine level on choices.

### Robustness

Above, we dedicated a whole section to derive conditions for the parameters of the learning rules Eqs 2 and 3 to guarantee correct (i.e. unbiased) estimation of payoffs and costs. We also pointed out that these conditions cannot be satisfied exactly even in theory; in fact, our own simulations throughout this paper yield parameter settings that significantly violate the conditions. The proposed biological implementation of the rules, certainly imperfect and subject to unpredictable influences, is yet less likely to feature parameters close to the constraint surface. How robust is the presented learning algorithm under parameter detuning? How much variation around the conditions can the rules take without breaking? Here, we first describe the effect of parameter detuning on the values to which Go and No-Go weights converge. Then, we argue that the algorithm will still produce useful results even under substantial detuning of the parameters.

We are interested in the coding of payoffs and costs after learning, and should therefore investigate the equilibrium values G* and N* of *G* and *N*. Those equilibrium values may be obtained via combination of the equilibrium values of Q* and S* given in Eqs 8 and 11:

$$\begin{array}{c} G^{*}=Q^{*}+S^{*}\approx c_{Q}q+c_{s}E|R-c_{q}q|\approx c_{Q}q+c_{s}s \end{array}$$ (27)

$$\begin{array}{c} -N^{*}=Q^{*}-S^{*}\approx c_{Q}q-c_{s}E|R-c_{q}q|\approx c_{Q}q-c_{s}s. \end{array}$$ (28)

Here, we assumed that the average spread around *c*_*Q*_ *q* is approximately equal to the average spread around *q*, which is a good approximation if the spread of a distribution is comparable to the mean. Next, we can use the relation of payoffs *p* and costs *n* to the statistics *q* and *s* of the reinforcement distribution they generate. These relations are given in Eqs 4 and 5; inverting and inserting those yields

$$\begin{array}{c} G^{*}\approx \frac{1}{2}(c_{Q}+c_{S})p-\frac{1}{2}(c_{Q}-c_{S})n \end{array}$$ (29)

$$\begin{array}{c} N^{*}\approx -\frac{1}{2}(c_{Q}-c_{S})p+\frac{1}{2}(c_{Q}+c_{S})n. \end{array}$$ (30)

We observe that as long as *c*_*Q*_ = *c*_*S*_, the Go and No-Go weights converge to the vicinity of values proportional to the payoffs and costs. Thus, as long as *c*_*Q*_ = *c*_*S*_, the payoffs and costs are encoded separately in the two pathways.

Expressed in terms of the elementary parameters *α*, λ and *ϵ*, and solved for *ϵ*, this condition becomes

$$\begin{array}{c} \epsilon =\sqrt{(2\lambda /\alpha {)}^{2}+1}-2\lambda /\alpha . \end{array}$$ (31)

A second solution of the condition exists; however, it yields *ϵ* < 0, which is biologically implausible. Hence, we ignore that second solution and focus our attention on Eq 31: if λ/*α* is very small (i.e. if decay is weak relative to learning), then *ϵ* approaches one, rendering the learning rules approximately linear. If, on the other hand, λ/*α* is very large (i.e. decay is very strong compared to learning), then *ϵ* approaches zero, rendering the learning rules maximally non-linear. This relationship between *ϵ* and λ is not surprising; in fact, we have seen in Fig 2 that decay is necessary to balance the unconstrained strengthening of the weighs that results from introducing the nonlinearity (compare Fig 2b and Fig 2c). Eq 31 makes this manifest: the stronger the nonlinearity (i.e. the closer *ϵ* gets to zero), the stronger the decay relative to learning–and vice versa.

Now, after investigating the effect of detuning on G* and N*, let us explore the effect of detuning on the thalamic activity *T*, which is the relevant output of our model as far as action selection is concerned. Substituting the above equations into the definition of thalamic activity in Eq 25 we obtain:

$$\begin{array}{c} T=p(\frac{1}{2}c_{Q}-\frac{1}{2}c_{S}+Dc_{S})-n(\frac{1}{2}c_{Q}+\frac{1}{2}c_{S}-Dc_{S}) \end{array}$$ (32)

When *c*_*Q*_ = *c*_*S*_ ≠ 1, the thalamic activity becomes scaled by a constant *c*_*S*_, but as this scaling constant is the same for all actions, the network can still select actions on the basis of payoffs and costs modulated by motivation signal *D*, in the same way as described in the previous subsection. Importantly, the effect of dopamine–to emphasize the payoff when increased, and emphasize the cost when decreased–is present as long as *c*_*S*_ > 0 even if *c*_*Q*_ ≠ *c*_*S*_. These signature effects of the proposed mechanism are thus robust even under significant detuning. However, the disadvantage of setting parameters such that *c*_*Q*_ ≠ *c*_*S*_ is that the dopaminergic motivation signal *D* would have a relatively smaller effect on changing the weighting between payoffs and costs; for example the payoffs or costs could no longer be ignored by setting *D* to its extreme values of 0 or 1. From this analysis, we may conclude that while action selection is quite robust under violation of the derived conditions, dopaminergic regulation works most effectively if the conditions are met approximately.

### An actor-critic variation

So far, we assumed that the outcome prediction is computed by the same striatal neurons that encode the payoffs and costs of actions. Only one network was involved: that which is responsible for the choice of action. We refer to such a network as ‘actor’ in the remainder of this exposition. In this section, we look at how the theory described above generalizes to the actor-critic framework [27]. That framework assumes that the outcome prediction is not computed by the actor, but by a separate group of striatal patch neurons called the ‘critic’. More formally, the purpose of that critic is to learn the value *V* of the current state.

One way to generalize our theory in this direction is to keep the actor network unaltered, while supplementing it with a similar critic network that learns by the very similar rules Eqs 2 and 3:

$$\begin{array}{c} \Delta G_{critic}(s)=\alpha f_{\epsilon }(\delta )-\lambda G_{critic}(s) \end{array}$$ (33)

$$\begin{array}{c} \Delta N_{critic}(s)=\alpha f_{\epsilon }(-\delta )-\lambda N_{critic}(s) \end{array}$$ (34)

The crucial difference between the actor and the critic is that the critic network is not selective for the action, but only for the situation (note that *G*_*critic*_(*s*) and *N*_*critic*_(*s*) depend on *s*, but not on *a*, as opposed to *G*_*actor*_ (*s*, *a*) and *N*_*actor*_ (*s*, *a*)). It thus learns the value of a situation irrespective of the actions chosen. Importantly, the critic is in charge of supplying the outcome predictions. Those predictions are compared to the actual outcomes to produce the outcome prediction errors *δ* from which both networks learn.

We take the state value to be encoded in the difference of *G*_*critic*_(*s*) and *N*_*critic*_(*s*): *V*_*critic*_(*s*) = 1/2(*G*_*critic*_(*s*) − *N*_*critic*_(*s*)). The change of the state value on each trial can be obtained by subtracting Eqs 33 and 34:

$$\begin{array}{c} \Delta V_{critic}(s)=\alpha \frac{\left(1+\varepsilon \right)}{2}\delta -\lambda V_{critic}(s) \end{array}$$ (35)

The prediction error *δ*—which teaches the actor as well—is the difference between the obtained reinforcement *r* and the reinforcement prediction by the critic:

$$\begin{array}{c} \delta =r-V_{critic}(s) \end{array}$$ (36)

What would be learned with that architecture? If the same action is selected on each trial, the actor will learn in exactly the same way as the critic. Then, the prediction error in the actor-critic model is the same as in the actor-only model described above, and the weights of the actor in the actor-critic model converge to exactly the same values as for the actor-only model. However, this reasoning does not seem to apply if more than one action is available: empirically, animals then select the actions that maximize their rewards in their own perception. In the process of learning, they will likely sample all available actions.

If such behavior generates input for an actor-critic model, the critic will integrate the experience of all those trials, and will thus represent a mixture of the expected reinforcements associated with the available actions. This generally interferes with correct learning of the payoffs and costs of the different actions. However, there is a caveat: one of the available actions will eventually prove most useful; as soon as the animal has determined that best action, it will select it in the majority of cases. That, in turn, forces the critic into mainly representing the expected reinforcement of this best action. As a final consequence, also payoff and cost of that best action are inferred correctly.

**Fig 9:**
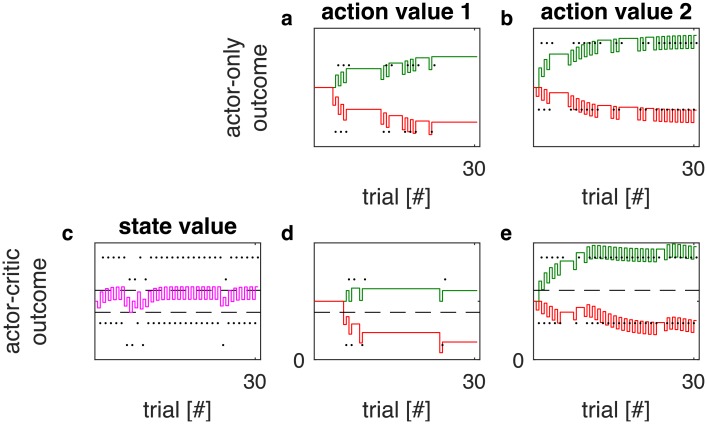
Actor-only in comparison with actor-critic learning. The columns labeled with ‘action value 1’ (panels a and d) and ‘action value 2’ (panels b and d) show the simulated evolution of the collective synaptic weights *G* and *N* of the actor network over 30 successive trials. The first row (panels a and b) shows the evolution of the actor network in an actor-only architecture, while the second row (panels d and e) provides the evolution of the actor in an actor-critic architecture. The weights *G* are drawn as solid green lines, the negative weights-*N* are drawn as solid red lines. The reinforcements obtained by choosing the respective actions are indicated by black dots. For the actor-critic simulations (second row), we additionally provide the evolution of the state value in panel c. There, the state value *V*_*critic*_ is represented by a solid purple line. The expected reinforcements of both actions are indicated by dashed horizontal lines. The parameter settings used in these simulations were *α* = 0.4, *ϵ* = 0.519, λ = 0.1013 and *β* = 0.9. The same set of parameters was used for both the actor-only and the actor-critic model.

We confirmed this empirically for the model specified above: in Fig 9, we present simulations of a task in which the subject must choose between two actions. Both actions reliably yield a constant cost followed by a constant payoff each time they are selected. One of the actions is unambiguously superior to the other: its payoff is larger and its cost is lower.

Both an actor-only model and an actor-critic model interacted with that task. On each trial, an action was selected by sampling from a softmax distribution over all available actions: the probability of choosing action *a* in situation *s* was proportional to exp (*βQ*(*s*, *a*)), where *Q*(*s*, *a*) = 1/2(*G*(*s*, *a*) − *N*(*s*, *a*)) was the action value, and *β* was the softmax temperature. Fig 9 shows the temporal evolution of the involved synaptic weights over the course of learning. Fig 9a and 9b depict the actor-only evolution of the weights *G* and *N* that encode the payoffs and costs of actions 1 and 2, respectively. For both actions, payoffs and costs are learned correctly. Learning is notably slower for action 1. This is easily explained: action 1 is the worse of the two options and thus chosen much less frequent. In contrast, the actor-critic driven evolution of the same weights presented in Fig 9d and 9e leads to a correct estimate of the payoff and cost only for the superior action 1. Learning is impaired for the inferior action 2, as anticipated in the qualitative discussion above. The state value, presented in Fig 9c, provides further confidence in the validity of that discussion: Instead of encoding a mixture of the values of all available actions, it converges to the value of the superior action, indicated by the higher of the two dashed lines.

What have we learned in this section? We set out to analyze an actor-critic formulation of our model, where the feedback signal that teaches the actor is computed by a different network called the critic. We found that our formulation (which is by no means the only possible one) enables the actor to learn accurate estimates of the payoffs and costs of the most advantageous action from the critic’s feedback. The payoffs and costs of the other actions were not estimated as accurately, which was due to a sampling bias towards more rewarding options. This does not necessarily compromise behavior–after all, one may trust the model to provide accurate information on the actions that are most frequently picked, and thus to be helpful in the majority of cases. However, we believe that a more sophisticated actor-critic variant of our model could conceivably provide good estimates of the payoffs and costs of all actions. The development of this improved actor-critic variant is left to future work; here we merely demonstrate that our model is not meant to compete with actor-critic models, but rather to complement them.

## Discussion

This article describes how the positive and negative consequences of actions can be separately learned on the basis of a single teaching signal encoding outcome prediction error. In this section, we relate the theory with data and other models, state experimental predictions, and highlight the directions in which the theory needs to be developed further.

### Relationship to experimental data

The model described in this paper was shown in simulations to avoid actions that require effort when the motivational signal was reduced. The unwillingness to make an effort for reward in dopamine-depleted state has also been observed in other paradigms: During a choice in a T-maze, dopamine-depleted animals were less likely to go to an arm with more pellets behind the barrier, but rather chose the arm with easily accessible but fewer pellets [28]. Parkinson’s patients were not willing to exert as much physical effort by squeezing a handle in order to obtain reward as healthy controls, especially if they were off medications [29]. These effects can be explained in an analogous way [8] by assuming that in the dopamine-depleted state the effort of crossing the barrier or squeezing a handle is weighted more, resulting in lower activity of thalamic neurons selective for this option. Both in OpAL and the model proposed here, reducing the dopamine level reduces the tendency to choose actions involving costs, and thus changes preferences.

**Fig 10:**
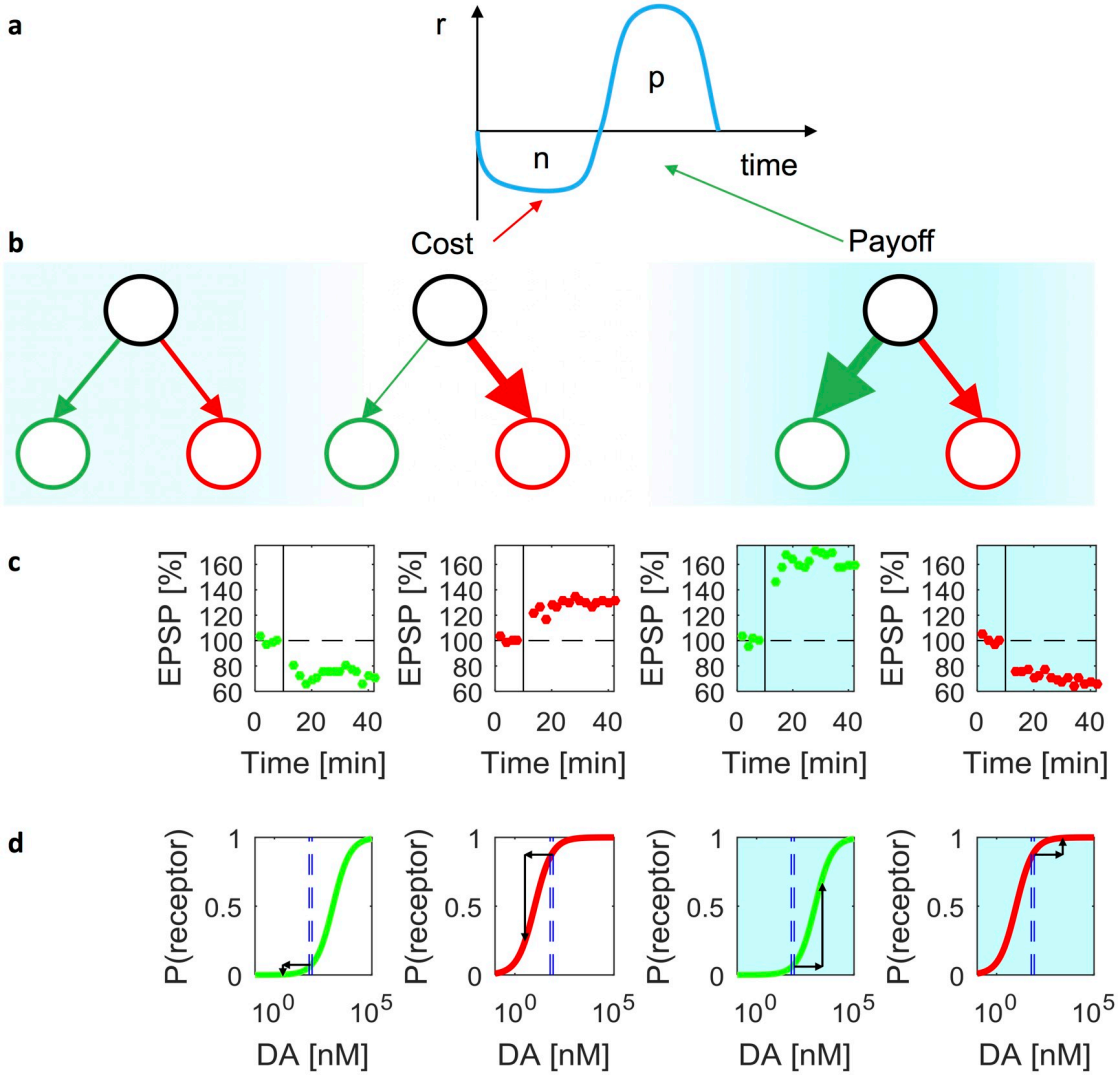
Relationship of learning rules to synaptic plasticity and receptor properties. (a) Instantaneous reinforcement *r* when an action with effort *n* is selected to obtain payoff *p*. (b) Cortico-striatal weights before the action, after performing the action, and after obtaining the payoff. Red and green circles correspond to striatal Go and No-Go neurons, and the thickness of the lines indicates the strength of synaptic connections. The intensity of the blue background indicates the dopaminergic teaching signal at different moments of time. (c) The average excitatory post-synaptic potential (EPSP) in striatal neurons produced by cortical stimulation as a function of time in the experiment reported in [11]. The vertical black lines indicate the time when synaptic plasticity was induced by successive stimulation of cortical and striatal neurons. The amplitude of EPSPs is normalized to the baseline before the stimulation indicated by horizontal dashed lines. The green and red dots indicate the EPSPs of Go and No-Go neurons respectively. Displays with white background show the data from experiments with rat models of Parkinson’s disease, while the displays with blue background show the data from experiments in the presence of corresponding dopamine receptor agonists. The four displays re-plot the data from Figures 3E, 3B, 3F and 1H in [11]. (d) Changes in dopamine receptor occupancy. The green and red curves show the probabilities of D1 and D2 receptor occupancies in a biophysical model [30]. The two dashed blue lines in each panel indicate the levels of dopamine in dorsal (60 nM) and ventral (85 nM) striatum estimated on the basis of spontaneous firing of dopaminergic neurons using the biophysical model [32]. Displays with white and blue backgrounds illustrate changes in receptor occupancy when the level of dopamine is reduced or increased respectively.

Let us now consider how the weight changes in our model relate to known data on synaptic plasticity in the striatum. Fig 10b illustrates the weight changes when an animal performs an action involving a cost *n* in order to obtain a payoff *p* (Fig 10a), e.g. pressing a lever in order to obtain a pellet. The direction of changes in *G* and *N* depending on the sign of *δ* are consistent with the changes of synaptic weights of Go and No-Go neurons observed at different dopamine concentrations. Fig 10c shows experimentally observed changes in synaptic strengths when the level of dopamine is low (displays with white background) and in the presence of agonists (blue background) [11]. Note that the directions of change match those in the corresponding displays above, in Fig 10b.

These directions of changes in striatal weights are also consistent with other models of the basal ganglia [8, 12], but the unique prediction of the rules described in this paper is that the increase in dopaminergic teaching signal should mainly affect changes in *G*, while the decrease in dopamine should primarily affect *N*. Thus, the dopamine receptors on the Go and No-Go neurons should be most sensitive to increases and decreases in dopamine level respectively. This matches the properties of these receptors. The D2 receptors on No-Go neurons have a higher affinity and therefore are sensitive to low levels of dopamine compared to D1 receptors on Go neurons [31]. This property is illustrated in Fig 10d where the green and red curves show the probabilities of D1 and D2 receptors being occupied as a function of dopamine concentration. The blue dashed lines indicate the levels of dopamine in the striatum predicted to result from the spontaneous firing of dopaminergic neurons [32]. At these levels, most D1 receptors are deactivated. Thus the D1 receptor activation will change when the dopamine goes up, but not when it goes down, as indicated by the black arrows. This is consistent with the stronger impact of positive prediction errors on the weight changes of the Go neurons implemented in Eq 2. By contrast, the D2 receptors are activated at baseline dopamine levels, so their activation is affected by the decreases in dopamine level but little by increases, in agreement with a stronger impact of positive prediction errors on the No-Go neurons implemented in Eq 3.

Our model further requires decay of relevant weights whenever prediction errors are absent. In terms of neural implementation, this translates into mild LTD resulting from co-activation of the pre- and post-synaptic cells at baseline dopamine levels. Recently, this effect has been observed at cortico-striatal synapses in vivo [33]: in anesthetized rats, presynaptic activity followed by postsynaptic activity caused LTD in the absence of induced dopaminergic response.

In summary, the plasticity rules allowing learning positive and negative consequences are consistent with the observed plasticity and the receptor properties.

Recently, there has been a debate concerning the fundamental concept of basal ganglia function, i.e. the relationship between the Go and No-Go neurons: on one hand they have the opposite effects on a tendency to make movements [2], but on the other hand they are co-activated during action selection [34]. The presented theory is consistent with both observations: It assumes that Go and No-Go neurons have opposite effects on movement initiation. But during action selection, the basal ganglia need to calculate the utility which combines information encoded by both populations, so may require their co-activation.

The proposed model assumes that while an animal makes an effort, the outcome prediction error should be negative, thus the dopamine level should decrease. However, at the time of lever pressing the system needs to be energized to perform a movement, so one could expect an increased level of dopamine. Furthermore, voltammetry studies measuring dopamine concentration in the striatum did not observe a decrease in dopamine level during lever pressing [35]. Nevertheless a recent study recording activity of single dopaminergic neurons that provided a better temporal resolution reported that dopaminergic neurons increased the activity before movement, and then decreased it below baseline during movement [32]. The increase before movement may be related with energizing system for movement, while the decrease during movement may be related with representing effort.

In addition to effort, other negative experiences lead to phasic decreases in dopaminergic activity as well: the unexpected experience of pain [36], aversive stimuli such as air puffs [37] and, for humans, monetary losses (literal costs) [38] all coincide with decreased activity of dopamine neurons. This supports the general idea that the No-Go pathway encode costs of all kinds.

### Experimental predictions

A direct test of the proposed model could involve the recording of the activity of Go and No-Go neurons (e.g. with photometry) during a task in which an animal learns the payoffs and costs associated with an action. Assuming that *G* and *N* are reflected in the activity of the Go and No-Go neurons while the animal evaluates an action (i.e. just before its selection), one could analyze the changes in the activity of Go and No-Go neurons across trials. One could compare if they follow the pattern predicted by the rules given in this paper, or rather by other rules proposed to describe learning in striatal neurons [7, 8, 14].

Just as the OpAL model [8], the theory proposes that the positive and negative consequences are separately encoded by the Go and No-Go neurons which are differentially modulated by dopamine. The theory predicts that agonists specific to just one of the striatal populations change the effect of consequences encoded by this population without changing the impact of the other population. For instance, a D1 antagonist would suppress the reception of dopamine in the direct pathway. There, dopamine increases activity. Hence, the D1 antagonist would diminish the impact of the direct pathway, and therefore of learned positive consequences, on choices. However, it would not change the impact of the indirect pathway, i.e. the impact of learned negative consequences. This prediction could be tested in an experiment involving choices between options with both payoff and cost. Consider, for instance, the decision between a neutral option (*p* = 1, *n* = 1) and a high-payoff option (*p* = 2, *n* = 1). Since a D1 antagonist decreases the impact of payoffs on decisions, it should decrease the preference for the high-payoff option. On the other hand, the avoidance of a high-cost option (*p* = 1, *n* = 2) over the neutral option should not be affected by the D1 antagonist, since it does not affect the impact of costs on decisions.

It could also be worthwhile to investigate whether changing the influence of positive and negative consequences on choice can not only be achieved by pharmacological manipulations, but also by changing a behavioral context such as hunger, or reward rate which has been shown to affect the average dopamine level [19].

The theory assumes that the synaptic plasticity rules include a decay term proportional to the value of the synaptic weights themselves. Decay terms are also present in other models of learning in basal ganglia [15, 39, 40]. This class of models predicts that the synaptic weights of striatal neurons which are already high increase less during potentiation than the smaller weights (an opposite prediction is made by the OpAL model [8], where the weights scale the prediction error in the update rule). This prediction could be tested by observing the Excitatory Post-Synaptic Currents (EPSCs) evoked at individual spines. The class of model including decay predicts that the spines with smaller evoked EPSCs before inducing plasticity should be more likely to potentiate.

### Relationship to other theories

The proposed model builds on the seminal work of Collins and Frank [8], who proposed that the Go and No-Go neurons learn the tendency to execute and inhibit movements, and how the level of dopamine changes the influence of the Go and No-Go pathways on choice. The key new feature of the present model is the ability to learn both payoffs and costs associated with a single action. We demonstrated above that when the model repeatedly selects an action resulting first in a cost and then in the payoff, *G* and *N*—under certain conditions that we specified—converge to the magnitudes of that payoff and cost. This is not so in the original OpAL model, as we shall show in a brief analysis.

Collins and Frank [8] demonstrated that when the environment is stationary and prediction error *δ* converges to zero, then the weights *G* and *N* in the OpAL model converge to bounded values. However, we will show that Go and No-Go weights converge to zero when an action that results first in a cost and then in the payoff is repeatedly selected.

The OpAL model is based on the actor-critic framework; hence, the prediction error is defined as in Eq 36. The weights of the critic are modified simply as Δ*V* = *αδ*. The weights of the actor are modified according to the following equations [8]:

$$\begin{array}{c} \Delta G=\alpha G\delta \end{array}$$ (37)

$$\begin{array}{c} \Delta N=-\alpha N\delta \end{array}$$ (38)

**Fig 11:**
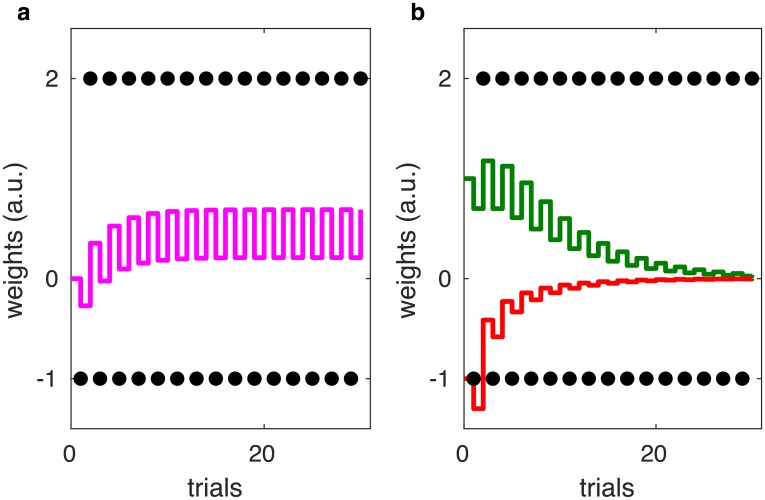
Changes in the weight *G* of the Go neurons, *N* of the No-Go neurons and *V* of the critic in the OpAL model over the course of simulations. (a) The purple line represents the evolving critic weight. The experienced reinforcements are indicated by black dots. (b) The actor weights, represented by a green and a red line respectively, were initialized to *G* = *N* = 1. Again, the black dots indicate the received reinforcements. The simulation was run with learning rate *α* = 0.3.

Fig 11 shows how the weights change in a simulation of the OpAL model. The weights of the critic approach a value close to the average of payoff and cost. Let us consider what happens in the model once the critic weight stops changing between trials (i.e. from ∼10th trial onward in Fig 11). The weight of the critic still changes within a trial, i.e. decreases when cost is incurred and increases after a payoff. This happens because the prediction error oscillates around 0, i.e. it is equal to *δ* = −*d* while incurring a cost and *δ* = *d* while receiving a payoff, where *d* is a constant. If so, let us consider how a Go weight changes within a trial. According to Eq 37 the weight changes as follows:

$$\begin{array}{c} G_{\text{after}\;\text{cost}}=G_{\text{before}\;\text{action}}-\alpha G_{\text{before}\;\text{action}}d \end{array}$$ (39)

$$\begin{array}{c} G_{\text{after}\;\text{payoff}}=G_{\text{after}\;\text{cost}}+\alpha G_{\text{after}\;\text{cost}}d \end{array}$$ (40)

Substituting Eq 39 into Eq 40 we obtain:

$$\begin{array}{cc} G_{\text{after}\;\text{payoff}} & =G_{\text{before}\;\text{action}}-\alpha G_{\text{before}\;\text{action}}d+\alpha \left(G_{\text{before}\;\text{action}}-\alpha G_{\text{before}\;\text{action}}d\right)d\\ & =G_{\text{before}\;\text{action}}-\alpha^{2}G_{\text{before}\;\text{action}}d^{2} \end{array}$$ (41)

We see that within a trial a Go weight decays proportionally to is value, resulting in an exponential decay across trials seen in Fig 11. Analogous calculations show that the No-Go weight decays in the same way. We conclude that the OpAL model is unable to estimate positive and negative consequences for actions which result in both payoffs and costs. It is worth noting that the decay of actor weights to zero demonstrated above is specific to the version of basal ganglia model proposed by Collins and Frank [8], but would not be present in another version of the model [39] where the learning rules include a special term preventing the weights from approaching zero. On the other hand, nothing in the above calculation depended on *G*, *N* and *V* updating at the same learning rate *α*–the derivation can be carried out in exactly the same way assuming *α*_*V*_ ≠ *α*_*N*_ ≠ *α*_*G*_. Hence, we may summarise that even such generalised OpAL models must fail to learn payoffs and costs of actions, irrespective of the specific parameter values unless further terms are added to the learning rules. Our analysis suggests that learning payoffs and costs can be enabled by different effective learning rates after positive versus negative feedback for Go and No-Go synapses, which in our model is achieved by setting *ϵ* < 1.

To interpret this result, note that we do not claim that the OpAL model is not capable of optimizing the policy. It is set up as a policy improving algorithm, and might even reflect the payoffs and costs of actions in the weights *G* and *N* in certain situations. However, as we have shown there is also situations in which OpAL is not able to encode the payoffs and costs. In contrast, we showed above the model presented in this paper does encode payoffs and costs in any situation, given a suitable set of parameters and enough time to learn.

The model described in this paper has been shown to account for the effects of dopamine depletion on the willingness to make effort, which have also been simulated with the OpAL model. To simulate the effects of dopamine depletion on the choice between an arm of a T-maze with more pellets behind a barrier and an arm with fewer pellets, [8] trained a model on three separate actions: eating in the left arm, eating in the right arm, and crossing a barrier. In this way, it was ensured that each action had just payoff or just cost, and the model could learn them. Subsequently, during choice, the model was deciding between a combination of two actions (e.g. crossing a barrier and eating in the left arm) and the other action. By contrast, the model proposed in this paper was choosing just between the two options available to an animal in an analogous task (Fig 6), because it was able to learn both payoffs and costs associated with each option. This is a useful ability, as most real-world actions have both payoffs and costs.

In the original paper introducing the plasticity rules [16], it was proposed that the rules allow the Go and No-Go neurons to encode reinforcement variability because when an action results in variable reinforcements, both *G* and *N* increase during learning. It was further proposed that the tonic level of dopamine controls the tendency to make risky choices, as observed in experiments [41], because it leads to emphasizing potential gains, and under-weighting potential losses. However, here it is proposed that the striatal learning rules primarily sub-serve a function more fundamental for survival, i.e. learning payoffs and costs of actions. From this perspective, the influence of dopamine level on the tendency to make risky choices arises as a by-product of a system primarily optimized to weight payoffs and costs according to the current motivational state.

### Directions for the future work

There are multiple directions in which the presented theory could be extended. For example, the theory has to be integrated with the models of action selection in the basal ganglia to describe how the circuit selects the action with the best trade-off of payoffs and costs. Furthermore, the theory may be extended to describe the dependence of the dopaminergic teaching signal on the motivational state. Learning experiments in which an animal may be deprived of physiologically required substances suggest that both terms in the outcome prediction error encoded by dopamine (i.e. the reinforcement and the expected outcome) are scaled by motivation [42]. It would be interesting to incorporate such scaling in our model, where the direct pathway, as well as the indirect pathway, contribute to the outcome estimate, which is then compared to the experienced reinforcement to compute the prediction error. If dopaminergic modulation is taken into account also at this stage, the dopaminergic motivation signal should affect the outcome estimate, and hence influence learning.

A limitation to our current model is the rudimentary form of the basal ganglia output, given in Eq 25. It is known that the effect of dopamine on the activity in the two pathways is not linear (as assumed in this paper), but exhibits saturation effects. The fact that the reception of dopamine is nonlinear plays a crucial role in the learning part of our model (the piecewise linear functions *f*_*ϵ*_ introduce exactly that nonlinearity), and could also be implemented at the decision-making stage, if the activity of Go and No-Go neurons (combined in Eq 25) depended nonlinearly on the dopamine level. In such more elaborate formulation, the fine-tuning of the baseline dopamine level then becomes critical. Including nonlinear effects of dopamine on activity during choice would allow studying interactions between learning and decision making, which would both be affected by the position of the baseline and the strength of the nonlinearity.

It is intriguing to ask whether the evaluation of actions combining separately encoded positive and negative consequences is also performed by areas beyond the basal ganglia. Indeed, positive and negative associations are encoded by different populations of neurons in the amygdala [43]. Moreover, an imaging study [44] suggests that costs and payoffs are predicted by the amygdala and the ventral striatum respectively, and ultimately compared in the prefrontal cortex. Furthermore, different cortical regions preferentially project to Go or No-Go neurons [45], raising the possibility that the positive and negative consequences are also encoded separately in the cortex. Therefore, it seems promising to investigate if similar plasticity rules could also describe learning beyond the basal ganglia.

## Materials and methods

During simulations of an experiment by Salamone et al. [22], the model received payoff *p*_chow_ = 1 for approaching the lab chow, and payoff *p*_pellet_ for choosing a pellet. The model was simulated in two conditions differing in the cost of choosing a pellet which was equal to *n*_pellet_ = 0 in the free-pellet condition, and to *n*_pellet_ = *n*_lever_ in a condition requiring lever pressing to obtain a pellet. There was no cost of choosing lab chow (*n*_chow_ = 0) in either condition.

For each condition, the model was simulated in two operational modes: in the control state, the coupling *κ*_*N*_ of dopamine to the D2-expressing neurons was fixed at *κ*_*N*_ = 1 during choice (making manifest the assumed fully functioning dopaminergic modulation in the control group). Conversely, in the state corresponding to the presence of the D2-antagonist haloperidol, *κ*_*N*_ was treated as a variable valued in [0, 1], now allowing for impaired dopaminergic regulation. The level of dopamine *D* was kept fixed at *D* = 0.5 throughout, assuming largely an unaltered baseline level for both groups.

For each condition and state, the behavior of *N*_*rats*_ was simulated. Each simulation consisted of 180 training and 180 testing trials (as each animal in the experiment of [22] was tested for 30 minutes, so 180 trials correspond to an assumption that a single trial took 10s). At the start of each simulation, the weights were initialized to *G*_pellet_ = *N*_pellet_ = *G*_chow_ = *N*_chow_ = 0.1. During each training trial, the model experienced choosing a pellet as well as approaching the lab chow. In detail, it received the cost *n*_pellet_, modified the weights *G*_pellet_ and *N*_pellet_, then received the payoff *p*_pellet_ and modified the weight again, and analogously for the lab chow. During each testing trial, the thalamic activity for each option was calculated from Eq 25), and Gaussian noise with standard deviation *σ* was added. An option with the highest thalamic activity was selected, and if this activity was positive, the action was executed, resulting in the corresponding cost and payoff and weight modification. If thalamic activity for both options was negative, no action was executed and no weights were updated.

The values of model parameters: *p*_pellet_, *n*_lever_, *κ*_*N*_, *σ* were optimized to match the choices made by the animals. In particular, for each set of parameters, the model was simulated *N*_*rats*_ = 100 times, and the average number of choices $c_{i,j,k}^{sim}$ of option *i* in dopamine state *j* and experimental condition *k* was computed. The mismatch with corresponding consumption in experiment $c_{i,j,k}^{exp}$ was quantified by a normalized summed squared error:

$$\begin{array}{c} Cost=\sum_{k=1}^{2}\sum_{j=1}^{2}\sum_{i=1}^{2}(\frac{c_{i,j,k}^{sim}}{Z_{k}^{sim}}-\frac{c_{i,j,k}^{exp}}{Z_{k}^{exp}}{)}^{2} \end{array}$$ (42)

In the above equation $Z_{k}^{dataset}$ is a normalization term equal to the total number of choices or consumption in a particular condition:

$$\begin{array}{c} Z_{k}^{dataset}=\sum_{j=1}^{2}\sum_{i=1}^{2}c_{i,j,k}^{dataset} \end{array}$$ (43)

The values of parameters minimizing the cost function were sought using the Simplex optimization algorithm implemented in Matlab, and the following values were found: *p*_pellet_ = 15.511751, *n*_lever_ = 14.510517, *κ*_*N*_ = 0.7507 and *σ* = 1.066246. Subsequently, the model with these optimized parameters was simulated with *N*_*rats*_ = 6, which was the number of animals tested by [22]. The resulting mean number of choices across animals are shown in Fig 8.

## References

[1] Redgrave P, Prescott TJ, Gurney K. The basal ganglia: a vertebrate solution to the selection problem? Neuroscience. 1999;89:1009–1023. 10362291 10.1016/s0306-4522(98)00319-4

[2] Kravitz AV, Freeze BS, Parker PR, Kay K, Thwin MT, Deisseroth K, et al Regulation of parkinsonian motor behaviours by optogenetic control of basal ganglia circuitry. Nature. 2010;466:622–626. 10.1038/nature09159 20613723 PMC3552484

[3] Smith Y, Beyan MD, Shink E, Bolam JP. Microcircuitry of the direct and indirect pathways of the basal ganglia. Neuroscience. 1998;86:353–388. 9881853 10.1016/s0306-4522(98)00004-9

[4] Surmeier DJ, Ding J, Day M, Wang Z, Shen W. D1 and D2 dopamine-receptor modulation of striatal glutamatergic signaling in striatal medium spiny neurons. Trends Neurosci. 2007;30:228–235. 10.1016/j.tins.2007.03.008 17408758

[5] Gurney K, Prescott TJ, Redgrave P. A computational model of action selection in the basal ganglia. I. A new functional anatomy. Biol Cybernetics. 2001;84:401–410. 10.1007/PL00007984 11417052

[6] Humphries MD, Khamassi M, Gurney K. Dopaminergic control of the exploration-exploitation trade-off via the basal ganglia. Frontiers in Neurosci. 2012;6:9 10.3389/fnins.2012.00009 PMC3272648 22347155

[7] Schroll H, Vitay J, Hamker H. Dysfunctional and compensatory synaptic plasticity in Parkinson’s disease European Journal of Neuroscience. 2014;39:688–702. 10.1111/ejn.12434 24313650

[8] Collins AG, Frank MJ. Opponent actor learning (OpAL): Modeling interactive effects of striatal dopamine on reinforcement learning and choice incentive. Psychol Rev. 2014;121:337–366. 10.1037/a0037015 25090423

[9] Schultz W, Dayan P, Montague PR. A neural substrate of prediction and reward. Science. 1997;275:1593–1599. 10.1126/science.275.5306.1593 9054347

[10] Eshel N, Tian J, Bukwich M, Uchida N. Dopamine neurons share common response function for reward prediction error. Nat Neurosci. 2016;19:479–486. 10.1038/nn.4239 26854803 PMC4767554

[11] Shen W, Flajolet M, Greengard P, Surmeier DJ. Dichotomous dopaminergic control of striatal synaptic plasticity. Science. 2008;321:848–851. 10.1126/science.1160575 18687967 PMC2833421

[12] Frank MJ, Seeberger LC, O’Reilly RC. By carrot or by stick: cognitive reinforcement learning in parkinsonism. Science. 2004;306:1940–1943. 10.1126/science.1102941 15528409

[13] Hong S, Hikosaka O. Dopamine-mediated learning and switching in cortico-striatal circuit explain behavioral changes in reinforcement learning. Frontiers Behav Neurosci. 2011;5 10.3389/fnbeh.2011.00015 PMC3065164 21472026

[14] Gurney KN, Humphries MD, Redgrave P. A new framework for cortico-striatal plasticity: behavioural theory meets in vitro data at the reinforcement-action interface. PLoS Biology. 2015;13:e1002034 10.1371/journal.pbio.1002034 25562526 PMC4285402

[15] Yttri EA, Dudman JT. Opponent and bidirectional control of movement velocity in the basal ganglia. Nature. 2016;533(7603):402–406. 10.1038/nature17639 27135927 PMC4873380

[16] Mikhael JG, Bogacz R. Learning reward uncertainty in the basal ganglia. PLoS Comput Biol. 2016;12:e1005062 10.1371/journal.pcbi.1005062 27589489 PMC5010205

[17] Niv Y. Cost, benefit, tonic, phasic. Ann NY Acad Sci. 2007;1104:357–376. 10.1196/annals.1390.018 17416928

[18] Howe M, Dombeck D. Rapid signalling in distinct dopaminergic axons during locomotion and reward. Nature. 2016;535:505–510. 10.1038/nature18942 27398617 PMC4970879

[19] Hamid AA, Pettibone JR, Mabrouk OS, Hetrick VL, Schmidt R, Vander Weele CM, et al Mesolimbic dopamine signals the value of work. Nat Neurosci. 2016;19:117–126. 10.1038/nn.4173 26595651 PMC4696912

[20] Berke JD. What does dopamine mean? Nat Neurosci. 2018 10.1038/s41593-018-0152-y 29760524 PMC6358212

[21] Maia TV, Frank MJ. An integrative perspective on the role of dopamine in schizophrenia. Biological psychiatry. 2017;81:52–66 10.1016/j.biopsych.2016.05.021 27452791 PMC5486232

[22] Salamone JD, Steinpreis RE, McCullough LD, Smith P, Grebel D, Mahan K. Haloperidol and nucleus accumbens dopamine depletion suppress lever pressing for food but increase free food consumption in a novel food choice procedure. Psychopharmacology. 1991;104:515–521. 10.1007/BF02245659 1780422

[23] Thurley K, Senn W, Lüscher HR. Dopamine increases the gain of the input-output response of rat prefrontal pyramidal neurons. J Neurophysiol. 2008;99:2985–2997. 10.1152/jn.01098.2007 18400958

[24] Hernández-López S, Tkatch T, Perez-Garci E, Galarraga E, Bargas J, Hamm H, Surmeier JD. D2 dopamine receptors in striatal medium spiny neurons reduce L-Type Ca2+ currents and excitability vía a novel PLC*β*1–IP3–calcineurin-signaling cascade. Journal of Neuroscience. 2000;20:8987–8995 10.1523/JNEUROSCI.20-24-08987.2000 11124974 PMC6773013

[25] Humphries MD, Lepora N, Wood R, Gurney K. Capturing dopaminergic modulation and bimodal membrane behaviour of striatal medium spiny neurons in accurate, reduced models Frontiers in computational neuroscience. 2009;26 10.3389/neuro.10.026.2009 PMC2791037 20011223

[26] Moyer JT, Wolf JA, Finkel LH. Effects of dopaminergic modulation on the integrative properties of the ventral striatal medium spiny neuron. Journal of neurophysiology. 2007;98:3731–3748 10.1152/jn.00335.2007 17913980

[27] Doya K. What are the computations of the cerebellum, the basal ganglia and the cerebral cortex? Neural Networks. 1999;12:961–974. 10.1016/S0893-6080(99)00046-5 12662639

[28] Salamone JD, Correa M, Yohn S, Cruz LL, San Miguel N, Alatorre L. The pharmacology of effort-related choice behavior: Dopamine, depression, and individual differences. Behav Process. 2016;127:3–17. 10.1016/j.beproc.2016.02.008 26899746

[29] Chong TTJ, Bonnelle V, Manohar S, Veromann KR, Muhammed K, Tofaris GK, et al Dopamine enhances willingness to exert effort for reward in Parkinson’s disease. Cortex. 2015;69:40–46. 10.1016/j.cortex.2015.04.003 25967086 PMC4533227

[30] Dreyer JK, Herrik KF, Berg RW, Hounsgaard JD. Influence of phasic and tonic dopamine release on receptor activation. J Neurosci. 2010;30:14273–14283. 10.1523/JNEUROSCI.1894-10.2010 20962248 PMC6634758

[31] Richfield EK, Penney JB, Young AB. Anatomical and affinity state comparisons between dopamine D1 and D2 receptors in the rat central nervous system. Neuroscience. 1989;30:767–777. 10.1016/0306-4522(89)90168-1 2528080

[32] Dodson PD, Dreyer JK, Jennings KA, Syed EC, Wade-Martins R, Cragg SJ, et al Representation of spontaneous movement by dopaminergic neurons is cell-type selective and disrupted in parkinsonism. P Natl Acad Sci USA. 2016;113:E2180–E2188. 10.1073/pnas.1515941113 PMC4839395 27001837

[33] Fisher SD, Robertson PB, Black MJ, Redgrave P, Sagar MA, Abraham WC, Reynolds JNJ. Reinforcement determines the timing dependence of corticostriatal synaptic plasticity in vivo. Nature communications. 2017;8(1):334 10.1038/s41467-017-00394-x 28839128 PMC5571189

[34] Cui G, Jun SB, Jin X, Pham MD, Vogel SS, Lovinger DM, et al Concurrent activation of striatal direct and indirect pathways during action initiation. Nature. 2013;494:238–242. 10.1038/nature11846 23354054 PMC4039389

[35] Syed EC, Grima LL, Magill PJ, Bogacz R, Brown P, Walton ME. Action initiation shapes mesolimbic dopamine encoding of future rewards. Nature Neurosci. 2016;19:34–36. 10.1038/nn.4187 26642087 PMC4697363

[36] Ungless MA, Magill PJ, Bolam JP. Uniform inhibition of dopamine neurons in the ventral tegmental area by aversive stimuli. Science. 2004;303:2040–2042. 10.1126/science.1093360 15044807

[37] Matsumoto M, Hikosaka O. Two types of dopamine neuron distinctly convey positive and negative motivational signals. Nature. 2009;459:837 10.1038/nature08028 19448610 PMC2739096

[38] Zaghloul KA, Blanco JA, Weidemann CT, McGill K, Jaggi JL, Baltuch GH, Kahana MJ. Human substantia nigra neurons encode unexpected financial rewards. Science. 2009;323:1496–1499. 10.1126/science.1167342 19286561 PMC2839450

[39] Franklin NT, Frank MJ. A cholinergic feedback circuit to regulate striatal population uncertainty and optimize reinforcement learning. Elife. 2015;4:e12029 10.7554/eLife.12029 26705698 PMC4764588

[40] Kato A, Morita K. Forgetting in Reinforcement Learning Links Sustained Dopamine Signals to Motivation. PLoS Comput Biol. 2016;12:e1005145 10.1371/journal.pcbi.1005145 27736881 PMC5063413

[41] Rutledge RB, Skandali N, Dayan P, Dolan RJ. Dopaminergic modulation of decision making and subjective well-being. J Neurosci. 2015;35:9811–9822. 10.1523/JNEUROSCI.0702-15.2015 26156984 PMC4495239

[42] Cone JJ, Fortin SM, McHenry JA, Stuber GD, McCutcheon JE, Roitman MF. Physiological state gates acquisition and expression of mesolimbic reward prediction signals. P Natl Acad Sci USA. 2016;113:1943–1948. 10.1073/pnas.1519643113 PMC4763767 26831116

[43] Namburi P, Beyeler A, Yorozu S, Calhoon GG, Halbert SA, Wichmann R, et al A circuit mechanism for differentiating positive and negative associations. Nature. 2015;520:675–678. 10.1038/nature14366 25925480 PMC4418228

[44] Basten U, Biele G, Heekeren HR, Fiebach CJ How the brain integrates costs and benefits during decision making. PNAS. 2010; 107:21767–21772 10.1073/pnas.0908104107 21118983 PMC3003102

[45] Wall NR, De La Parra M, Callaway EM, Kreitzer AC. Differential innervation of direct-and indirect-pathway striatal projection neurons. Neuron. 2013;79:347–360. 10.1016/j.neuron.2013.05.014 23810541 PMC3729794

